# Response of free-headed segmental piles with mechanical joints to lateral loading

**DOI:** 10.1038/s41598-026-36214-w

**Published:** 2026-01-22

**Authors:** Tao Liu, Qunqun Zhang, Chuanzhi Sun, Jingsheng Cheng, Yong Wu

**Affiliations:** 1 School of Civil Engineering and Architecture, Suqian University, Suqian, 223800 China; 2 Jiangsu Province Engineering Research Center of Prefabricated Building and Intelligent Construction, Suqian University, Suqian, 223800 China; 3 China Chemical East China Construction Co., Ltd., Suzhou, 215000 China; 4 Suqian Urban Construction Investment (Group) Co., Ltd., Suqian, 223800 China; 5 The Third Construction Co., Ltd., of China Construction Eighth Engineering Division, Nanjing, 210000 China

**Keywords:** *M*-method, Lateral loading, Mechanically-jointed piles, Numerical simulations, Mechanical responses, Engineering, Materials science

## Abstract

Segmental piles with mechanical joints(hereinafter, mechanically-jointed piles), as an improved pile type, have been widely adopted in construction projects. Due to their structural differences from conventional single piles, their mechanical responses diverge significantly, particularly under lateral loading. Gaps form at the mechanical joint between two single pile segments in mechanically-jointed piles, amplifying distinctions in mechanical response compared to conventional piles. To investigate the mechanical behavior of mechanically-jointed piles under lateral loading, this study develops a calculation theory for mechanical response based on the *m*-method—a standard approach for conventional single piles. The theory’s feasibility is validated via numerical simulations. Results indicate that numerical simulation align closely with *m*-method calculations: pile head displacement error is 4.8%, rotation error 6.2%, maximum bending moment error 24.9%, and maximum shear force error 8.2%. Comparative analysis of conventional single piles and mechanically-jointed piles with free ends reveals that under lateral loading, mechanically-jointed piles exhibit approximately 30% larger pile head displacement and approximately 55% greater rotation than conventional piles, indicating reduced deformation resistance. However, the results indicate that the mechanically-jointed pile can effectively reduce the maximum bending moment in the pile shaft. This reduction suggests a potential for optimizing the pile design and enhancing its lateral resistance performance under certain conditions.

## Introduction

Mechanically-jointed piles employ pre-set holes and interlocking connectors to enable rapid and reliable on-site connections through compression^1,2^. Compared to conventional monolithic piles, this system enhances construction efficiency and sustainability by providing robust pile-to-pile connections and simplifying pile-to-cap details.

Research on mechanically-jointed piles has primarily focused on the tensile, flexural, and shear performance of the joints themselves^3–7^. Under vertical loading, these piles behave similarly to conventional piles if joint integrity is maintained. However, under lateral loading, the joints introduce additional rotations, leading to discontinuous stress-deformation profiles and abrupt changes at the interfaces. Consequently, lateral bearing capacity calculations must account for these rotational discontinuities. While the behavior of laterally loaded piles has been extensively studied^8–15^, investigations addressing the specific influence of mechanical joints remain limited^16–18^. For instance, Gao et al.^17^ developed a theoretical solution for fixed-base mechanically-jointed piles using the *m*-method, establishing a basis for this pile type under restrained end conditions.

The behavior of mechanically-jointed piles under free-head conditions—where the pile tip provides negligible rotational restraint—remains poorly understood, despite its significant practical relevance. Theoretically, the free-head condition presents a more rigorous benchmark for analytical methods such as the *m*-method, testing their ability to to handle the interaction between an internal structural discontinuity ( joint rotation) and a free-boundary condition at the base. Practically, this scenario corresponds to critical field conditions where tip restraint is compromised, such as piles founded on soft soil strata, thin roofs over karst cavities, or in scour-prone environments^19–22^. The absence of specific design guidance for mechanically-jointed piles in these geotechnical contexts underscores the need for focused investigation.

To address this gap, this study aims to: (1) extend the *m*-method framework to model the response of free-headed segmental piles with a rotational mechanical joint; (2) validate the proposed theoretical model through comprehensive 3D finite element analysis; (3) clarify the distinct mechanical response and load-transfer mechanisms by comparing such piles with both conventional single piles and fixed-base connected piles. A simplified mechanical model based on the Winkler foundation beam theory and the *m*-method is established, with governing equations solved via the power series method. The findings aim to provide a theoretical foundation for analyzing and designing mechanically-jointed piles in conditions where base restraint cannot be assured.

## Basic theories

### Winkler foundation beam model

**Fig. 1:**
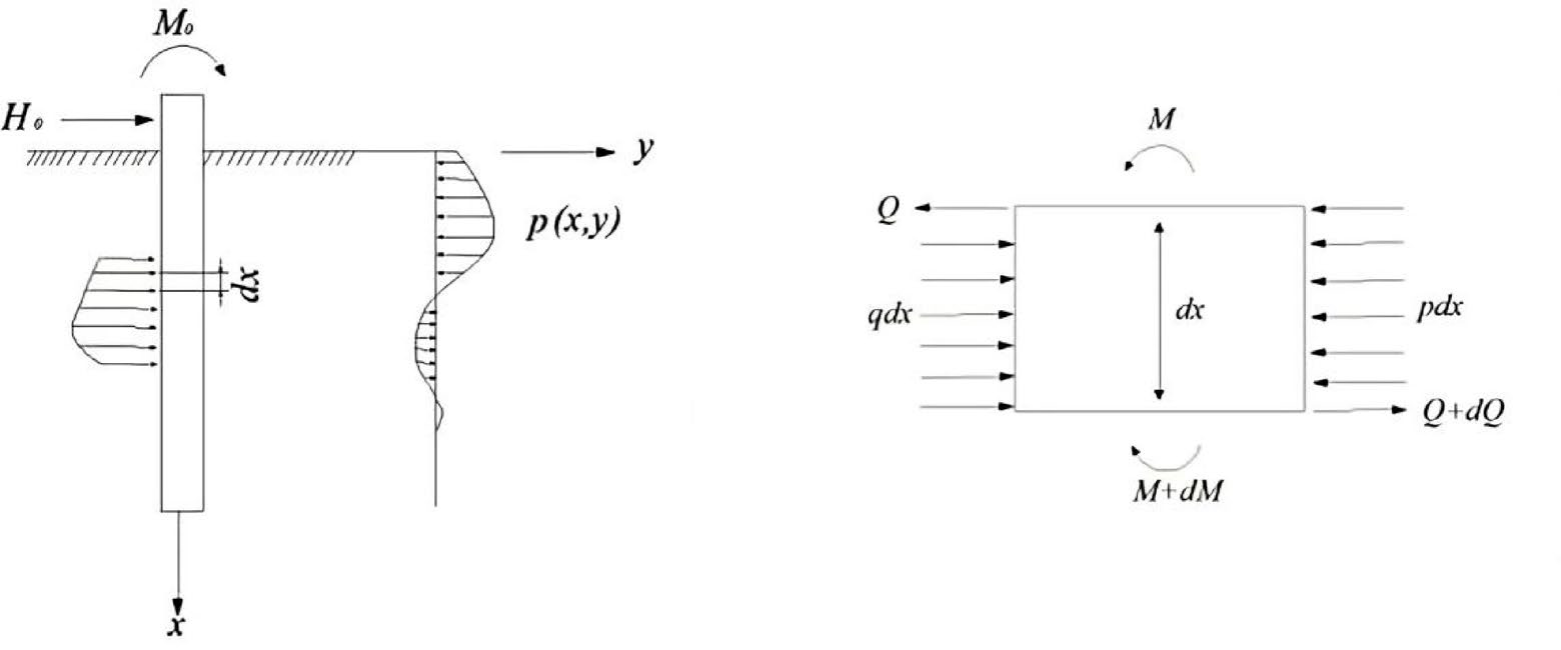
Schematic of the pile–soil interaction under lateral loading based on the *Winkler* foundation beam model.

In the study of pile-soil interaction, the basic and important problem is to correctly describe the mechanical state of the pile side soil and reasonably consider the influence of the pile side soil on the mechanical behavior of the pile. At present, most of them are Winkler foundation beam model ^23,24^ based on the Winkler hypothesis, the basic principle is to regard the pile as an elastic foundation beam placed in the soil, and to simulate the dynamic impedance of the pile side soil to the pile with springs and dampers that are continuously distributed and independent of each other, Fig. 1 demonstrates the force distribution within the model under lateral loading.

Within the framework of the Winkler foundation beam theory, Eq. (1) presents the deflection differential equation for an elastic long pile derived from static equilibrium principles under lateral loading.

1 $$EI\frac{{{\mathrm{d}}^{4} y}}{{dz^{4} }} + b_{0} P(z,y) = 0$$

where *E* = elastic modulus (*kPa*), *I* = moment of inertia (*m*^4^), *b*₀ = pile width (*m*), *y* = lateral displacement (*m*), *z* = depth (m), *P(z,y)* = soil resistance per unit area (*kN*/*m*^2^). Per *Winkler*’s assumption:

2 $$P(z,y) = k(z)y$$

where *k(z)* = coefficient of horizontal subgrade reaction (*kN*/*m*^3^).

### *m*-method

The*m*-method^25–28^ defines *k(z)* as:

3 $${\mathrm{k}}(z) = mz$$

where *m* = proportionality coefficient of horizontal subgrade reaction (*kN*/*m*^4^), typically determined empirically or via codes (e.g.,*JGJ 94-2008*^27^). For the fill soil considered in the numerical model and theoretical calculations, the adopted *m* value is 4000 kN/m^4^, as listed in Table 1.Substituting (3) into (1) yields:

4 $$\frac{{{\mathrm{d}}^{4} y}}{{dz^{4} }} + \alpha^{5} {\mathrm{z}}y = 0$$

where $\alpha = \sqrt[5]{{\frac{{{\mathrm{m}}b_{0} }}{{EI}}}} =$ pile deformation coefficient (1/m).

**Table 1:** Physical and mechanical parameters of soil layers.

Soil layer	Density ρ (kg/m^3^)	Elastic Modulus E (MPa)	Poisson’s ratio ν	Horizontal subgrade coefficient *m* (kN/m^4^)	Mohr–Coulomb model parameters	
					Friction angle φ(°)	Cohesion*c*(kPa)
Fill	2000	16	0.3	4000	24	7

### Power series solution method

#### Solution of the governing equation

Equation (4), a fourth-order differential equation with variable coefficients, is solved using the power series method to achieve the required computational accuracy. The solution takes the form:

5 $$y(z) = a_{0} f_{0} (z) + a_{1} f_{1} (z) + a_{2} f_{2} (z) + a_{3} f_{3} (z)$$

where:

6 $$\left\{ \begin{gathered} f_{0} (z) = 1 + \sum\nolimits_{k = 1}^{\infty } {( - 1)^{k} \frac{{T_{5}^{4} (K)}}{(5K)!}(\alpha z)^{5k} } \hfill \\ f_{1} (z) = z + \sum\nolimits_{k = 1}^{\infty } {( - 1)^{k} \frac{{T_{5}^{3} (K)}}{\alpha (5K + 1)!}(\alpha z)^{5k + 1} } \hfill \\ f_{2} (z) = z^{2} + \sum\nolimits_{k = 1}^{\infty } {( - 1)^{k} \frac{{2T_{5}^{2} (K)}}{{\alpha^{2} (5K + 2)!}}(\alpha z)^{5k + 2} } \hfill \\ f_{3} (z) = z^{3} + \sum\nolimits_{k = 1}^{\infty } {( - 1)^{k} \frac{{6T_{5}^{2} (K)}}{{\alpha^{3} (5K + 3)!}}(\alpha z)^{5k + 3} } \hfill \\ \end{gathered} \right.$$

The stepwise product operator is defined as:

7 $$T_{{\mathrm{m}}}^{n} (k) = (mk - n)[m(k - 1) - n]...(m - n)\quad (m > n)$$

This denotes sequential multiplication starting from *mk − n*, decrementing by *m* at each step until *m − n*. For example: $T_{5}^{4} (4) = 16 \times 11 \times 6 \times 1$, Constants *a*_0_ -*a*_3_ are determined by boundary conditions.

#### Dimensionless formulation

Implementing pile-head boundary conditions (z = 0):

- Displacement $${\mathrm{y}}_{0}$$
- Rotation $$\varphi_{0}$$
- Bending moment $${\mathrm{M}}_{0}$$
- Shear force $${\mathrm{Q}}_{0}$$

in Eq. (5) yields:

8 $$\left\{ \begin{gathered} y(z) = y_{0} A_{1} + \frac{{\varphi_{0} }}{\alpha }B_{1} + \frac{{M_{0} }}{{\alpha^{2} EI}}C_{1} + \frac{{Q_{0} }}{{\alpha^{3} EI}}D_{1} \hfill \\ \varphi (z) = \alpha y_{0} A_{1} + \varphi_{0} B_{1} + \frac{{M_{0} }}{\alpha EI}C_{1} + \frac{{Q_{0} }}{{\alpha^{2} EI}}D_{1} \hfill \\ M(z) = \alpha^{2} y_{0} EIA_{1} + \alpha \varphi_{0} B_{1} + \alpha \varphi_{0} EIC_{1} + \frac{{Q_{0} }}{\alpha }D_{1} \hfill \\ Q(z) = \alpha^{3} y_{0} EI + \alpha^{2} \varphi_{0} B_{1} + \alpha^{2} \varphi_{0} EIC_{1} + Q_{0} D_{1} \hfill \\ \end{gathered} \right.$$

The deformation coefficient $\alpha$(units: *m*⁻^1^) enables normalized depth $\overline{{\mathrm{z}}} = \alpha z$ (dimensionless). The terms $\alpha {\mathrm{y}}$,$M/\alpha EI$, and $Q/\alpha^{2} EI$ are likewise dimensionless.

Define normalized parameters:

9 $$\left\{ \begin{gathered} \overline{{\mathrm{y}}} = \alpha y \hfill \\ \overline{\varphi } = \varphi \hfill \\ \overline{M} = \frac{M}{\alpha EI} \hfill \\ \overline{Q} = \frac{Q}{{\alpha^{2} EI}} \hfill \\ \end{gathered} \right.$$

Equations (8)–(9) condense to the unified matrix form:

10 $$\left[ \begin{gathered} \overline{y(z)} \hfill \\ \overline{\varphi (z)} \hfill \\ \overline{M(z)} \hfill \\ \overline{Q(z)} \hfill \\ \end{gathered} \right] = \left[ \begin{gathered} A_{1} {\kern 1pt} {\kern 1pt} {\kern 1pt} {\kern 1pt} {\kern 1pt} B_{1} {\kern 1pt} {\kern 1pt} {\kern 1pt} {\kern 1pt} {\kern 1pt} C_{1} {\kern 1pt} {\kern 1pt} {\kern 1pt} D_{1} \hfill \\ A_{2} {\kern 1pt} {\kern 1pt} {\kern 1pt} {\kern 1pt} {\kern 1pt} B_{2} {\kern 1pt} {\kern 1pt} {\kern 1pt} {\kern 1pt} {\kern 1pt} C_{2} {\kern 1pt} {\kern 1pt} {\kern 1pt} D_{2} \hfill \\ A_{3} {\kern 1pt} {\kern 1pt} {\kern 1pt} {\kern 1pt} {\kern 1pt} B_{3} {\kern 1pt} {\kern 1pt} {\kern 1pt} {\kern 1pt} {\kern 1pt} C_{3} {\kern 1pt} {\kern 1pt} {\kern 1pt} D_{3} \hfill \\ A_{4} {\kern 1pt} {\kern 1pt} {\kern 1pt} {\kern 1pt} {\kern 1pt} B_{4} {\kern 1pt} {\kern 1pt} {\kern 1pt} {\kern 1pt} {\kern 1pt} C_{4} {\kern 1pt} {\kern 1pt} {\kern 1pt} D_{4} \hfill \\ \end{gathered} \right]{\kern 1pt} {\kern 1pt} {\kern 1pt} {\kern 1pt} \left[ \begin{gathered} \overline{{y_{0} }} \hfill \\ \overline{{\varphi_{0} }} \hfill \\ \overline{{M_{0} }} \hfill \\ \overline{{Q_{0} }} \hfill \\ \end{gathered} \right]$$

Coefficients $A_{1}$,$B_{1}$,$C_{1}$,$D_{1}$ depend on normalized depth $\overline{{\mathrm{z}}}$:

11 $$\left\{ \begin{gathered} A_{1} = {\mathrm{f}}_{0} (z) = 1 + \sum\nolimits_{k = 1}^{\infty } {( - 1)^{k} \frac{{T_{5}^{4} (K)}}{(5K)!}(\alpha z)^{5k} } \hfill \\ B_{1} = \alpha {\mathrm{f}}_{1} (z) = \alpha z + \sum\nolimits_{k = 1}^{\infty } {( - 1)^{k} \frac{{T_{5}^{3} (K)}}{(5K + 1)!}(\alpha z)^{5k + 1} } \hfill \\ C_{1} = \frac{{\alpha^{2} }}{2}{\mathrm{f}}_{2} (z) = \frac{{(\alpha z)^{2} }}{2} + \sum\nolimits_{k = 1}^{\infty } {( - 1)^{k} \frac{{T_{5}^{2} (K)}}{(5K + 2)!}(\alpha z)^{5k + 2} } \hfill \\ D_{1} = \frac{{\alpha^{3} }}{6}{\mathrm{f}}_{3} (z) = \frac{{(\alpha z)^{3} }}{6} + \sum\nolimits_{k = 1}^{\infty } {( - 1)^{k} \frac{{T_{5}^{2} (K)}}{(5K + 3)!}(\alpha z)^{5k + 3} } \hfill \\ \end{gathered} \right.$$

Subsequent coefficients derive from sequential differentiation and normalization:

**Table:** 

Coefficient set	Derivation procedure
$A_{2}$,$B_{2}$,$C_{2}$,$D_{2}$	$\frac{1}{\alpha }\frac{{\mathrm{d}}}{dz}\{ A_{1} ,B_{1} ,C_{1} ,D_{1} \}$
$A_{3}$,$B_{3}$,$C_{3}$,$D_{3}$	$\frac{1}{\alpha }\frac{{\mathrm{d}}}{dz}\{ A_{2} ,B_{2} ,C_{2} ,D_{2} \}$
$A_{4}$,$B_{4}$,$C_{4}$,$D_{4}$	$\frac{1}{\alpha }\frac{{\mathrm{d}}}{dz}\{ A_{3} ,B_{3} ,C_{3} ,D_{3} \}$

Mathematically expressed as:

12 $$\left\{ \begin{gathered} A_{2} = \sum\nolimits_{k = 1}^{\infty } {( - 1)^{k} \frac{{T_{5}^{4} (K)}}{(5K - 1)!}(\alpha z)^{5k - 1} } \hfill \\ B_{2} = 1 + \sum\nolimits_{k = 1}^{\infty } {( - 1)^{k} \frac{{T_{5}^{3} (K)}}{(5K)!}(\alpha z)^{5k} } \hfill \\ C_{2} = \alpha {\mathrm{z}} + \sum\nolimits_{k = 1}^{\infty } {( - 1)^{k} \frac{{T_{5}^{2} (K)}}{(5K + 1)!}(\alpha z)^{5k + 1} } \hfill \\ D_{2} = \frac{{(\alpha z)^{2} }}{2} + \sum\nolimits_{k = 1}^{\infty } {( - 1)^{k} \frac{{T_{5}^{2} (K)}}{(5K + 2)!}(\alpha z)^{5k + 2} } \hfill \\ \end{gathered} \right.$$

13 $$\left\{ \begin{gathered} A_{3} = \sum\nolimits_{k = 1}^{\infty } {( - 1)^{k} \frac{{T_{5}^{4} (K)}}{(5K - 2)!}(\alpha z)^{5k - 2} } \hfill \\ B_{3} = \sum\nolimits_{k = 1}^{\infty } {( - 1)^{k} \frac{{T_{5}^{3} (K)}}{(5K - 1)!}(\alpha z)^{5k - 1} } \hfill \\ C_{3} = 1 + \sum\nolimits_{k = 1}^{\infty } {( - 1)^{k} \frac{{T_{5}^{2} (K)}}{(5K)!}(\alpha z)^{5k} } \hfill \\ D_{3} = \alpha {\mathrm{z}} + \sum\nolimits_{k = 1}^{\infty } {( - 1)^{k} \frac{{T_{5}^{2} (K)}}{(5K + 1)!}(\alpha z)^{5k + 1} } \hfill \\ \end{gathered} \right.$$

14 $$\left\{ \begin{gathered} A_{4} = \sum\nolimits_{k = 1}^{\infty } {( - 1)^{k} \frac{{T_{5}^{4} (K)}}{(5K - 3)!}(\alpha z)^{5k - 3} } \hfill \\ B_{4} = \sum\nolimits_{k = 1}^{\infty } {( - 1)^{k} \frac{{T_{5}^{3} (K)}}{(5K - 2)!}(\alpha z)^{5k - 2} } \hfill \\ C_{4} = \sum\nolimits_{k = 1}^{\infty } {( - 1)^{k} \frac{{T_{5}^{2} (K)}}{(5K - 1)!}(\alpha z)^{5k - 1} } \hfill \\ D_{4} = 1 + \sum\nolimits_{k = 1}^{\infty } {( - 1)^{k} \frac{{T_{5}^{2} (K)}}{(5K)!}(\alpha z)^{5k} } \hfill \\ \end{gathered} \right.$$

### Mechanical model and fundamental assumptions

**Fig. 2:**
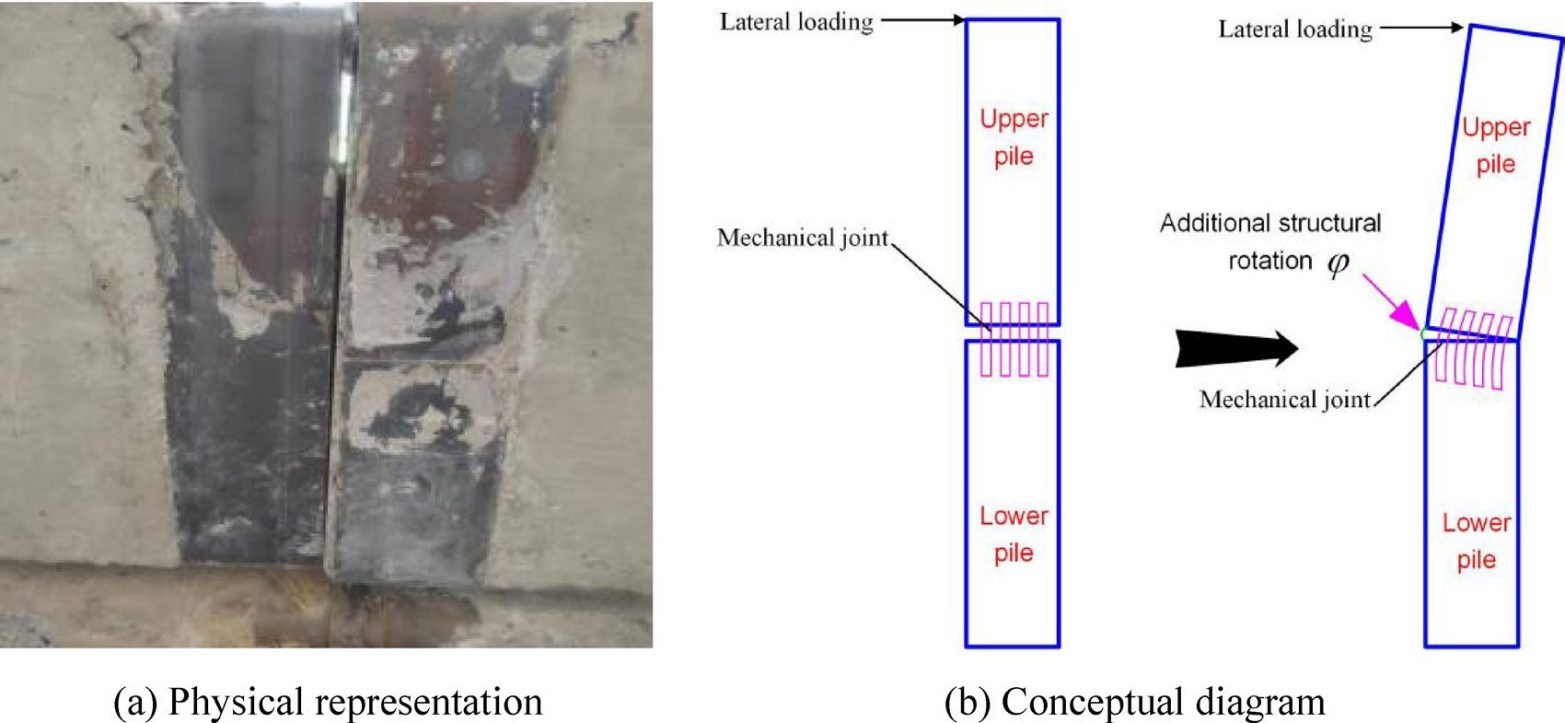
Structural rotation at the mechanical joint in a segmented pile under lateral loading.

Under lateral loading, mechanically-jointed piles exhibit non-uniform deformation distinct from the conventional long pile. An additional structural rotation $\varphi$ develops at the mechanical joint, as illustrated in Fig. 2.

Progressive loading drives the pile through three distinct mechanical phases. Displacement and internal force discontinuities induced by $\varphi$ necessitate segmented mechanical modeling of upper and lower pile segments. Prior to $\varphi$ reaching its limit $\varphi^{0}$, force transfer to the lower segment is neglected. Differential equations with phase-specific boundary conditions govern each stage:

1. Gap Development Stage

Application of lateral loading at the pile head induces displacement and rotation. The mechanically-jointed pile is modeled as an idealized plastic hinge permitting rotation without horizontal displacement at the upper segment base. The lower segment remains fixed and mechanically inactive.

(2) Critical State

As loading increases during gap development, $\varphi$ at the upper segment base attains the structurally limited value $\varphi^{0}$. This defines the first critical state, with boundary conditions yielding the corresponding critical load combination.

(3) Collaborative Stage

Post-critical loading increases rotation at the upper segment base while maintaining $\varphi = \varphi^{0}$. The lower segment initiates rotation driven by the mechanical joint, enabling moment and shear transfer. Displacement equations for both segments are solved simultaneously via geometric compatibility and force equilibrium at the joint.

(4) Determination of the Joint Rotation Limit ($\varphi^{0}$)

The mechanical joint is idealized as a hinge with a fixed rotation limit of $\varphi^{0} = 1000 \times 10^{ - 5} {\mathrm{r}}$. This value is derived from a simplified geometric analysis of the specific interlocking connector type under consideration. It estimates the critical angle at which the initially separated components of the joint (e.g., mating surfaces, locking elements) come into substantial contact, thereby initiating significant moment transfer. The estimation primarily considers the designed geometric clearance and the engagement depth of the interlocking features. This value serves as a representative and necessary parameter to enable the analytical formulation of the two-stage mechanical behavior (gap development and collaborative stages). It is acknowledged that the precise value may vary with specific connector designs and material tolerances. A comprehensive sensitivity analysis of how this parameter influences the global response, while beyond the scope of this foundational study, is recommended as an important avenue for future research to refine the model’s applicability (see Section “Future work”).

## Derivation of mechanical response equations

The extension of the classical m-method and Winkler foundation beam theory, originally formulated for continuous monolithic piles, to a segmented system with a mechanical joint constitutes a fundamental assumption of this analytical model. This approach implicitly presumes that the soil reaction on either side of the joint remains governed by the local pile displacement and is not significantly influenced by the discontinuity itself (i.e., independent spring action is maintained). The validity of this assumption is considered reasonable under the specific scope of this study, which focuses on the serviceability limit state characterized by small deformations and elastic soil response. Under these conditions, the physical gap opening at the joint due to its rotation is typically negligible compared to the pile diameter. Consequently, the disturbance to the continuous stress field in the surrounding soil is limited, and the independent spring idealization of the Winkler model provides a workable first-order approximation for deriving the pile’s global response. It is acknowledged, however, that this assumption may require refinement for analyses involving large deformations, plastic soil yielding adjacent to the joint, or specific joint geometries that induce significant soil flow or arching effects. This inherent limitation of the current model is explicitly noted in the concluding section.

### Gap development phase

**Fig. 3:**
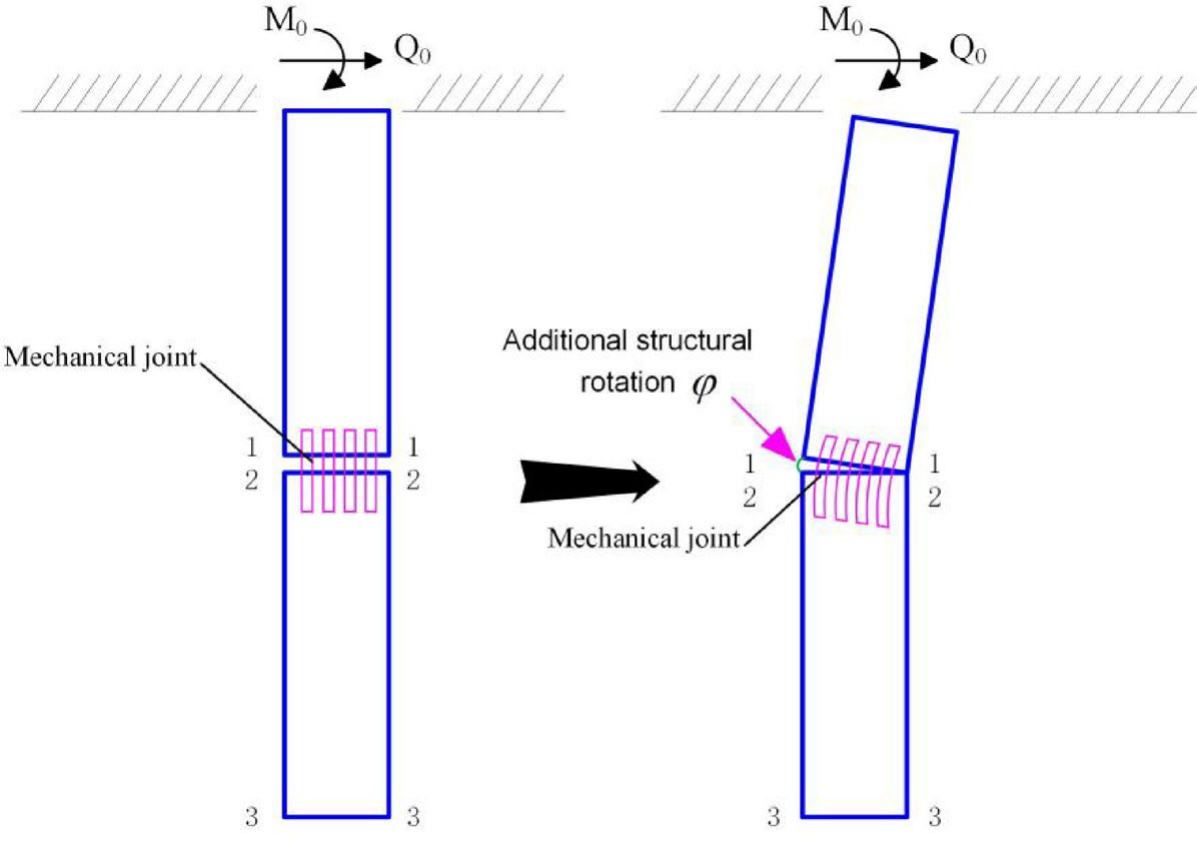
Deformation pattern of the segmented pile during the gap-development stage.

Before the structural rotation $\varphi$ reaches its limit $\varphi^{0}$ , the lower pile segment is assumed undeformed and stress-free. Thus, only the upper segment’s response is analyzed. The deformation pattern is shown in Fig. 3.

#### Governing equation for upper segment

**Fig. 4:**
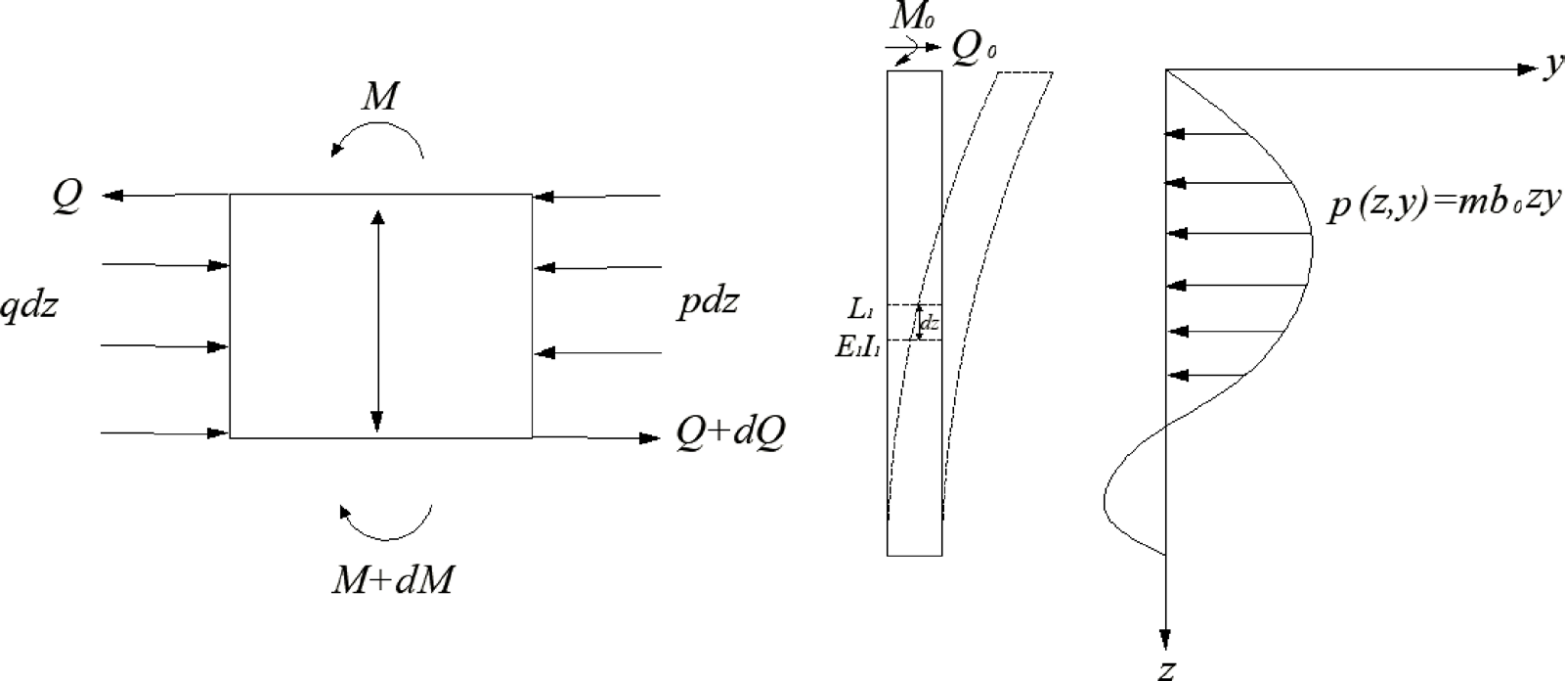
Schematic of the internal forces acting on the upper segment during the gap-development stage.

The upper segment (length $L = L_{1}$ ) interacts with surrounding soil under lateral forces and moments (Fig. 4).

Based on the*m*-method, its deflection equation is:

15 $$\frac{{{\mathrm{d}}^{4} y}}{{dz^{4} }} + \alpha_{1}^{5} {\mathrm{z}}y = 0$$

where:

- $$\alpha_{1} = \sqrt[5]{{\frac{{mb_{01} }}{{E_{1} I_{1} }}}}$$: Deformation coefficient (m⁻1)
- ${\mathrm{b}}_{01}$: Effective width (m) per *JTG 3363-2019* ^29^
- $E_{1} I_{1} = \beta E_{c} I$: Flexural rigidity (kN•m^2^) with *β* = 0.85 for RC piles.

Boundary conditions (free head, hinged base):

16 $$\left\{ \begin{gathered} \left\{ \begin{gathered} M({\mathrm{z}} = 0) = 0 \hfill \\ Q(z = 0) = 0 \hfill \\ \end{gathered} \right.,{\text{Free - head }} \hfill \\ \left\{ \begin{gathered} y(z = L_{1} ) = 0 \hfill \\ \varphi (z = L_{1} ) = \varphi_{1} \hfill \\ \end{gathered} \right.,Hin{\text{ge - base}} \hfill \\ \end{gathered} \right.$$

The base moment $M_{1}$ corresponding to rotation $\varphi_{1}$ is:

17 $$M_{1} = \int\limits_{{A_{1} }} {{\mathrm{zd}}N_{z} } = - \int\limits_{{A_{1} }} {{\mathrm{z}}^{2} \varphi_{1} } C_{{L_{1} }} dA_{1} = - \varphi_{1} C_{{L_{1} }} \int\limits_{{A_{1} }} {{\mathrm{z}}^{2} } dA_{1} = - \varphi_{1} C_{{L_{1} }} I_{{L_{1} }}$$

where $A_{1}$ = Cross-sectional area(m^2^), $I_{{L_{1} }}$ = Moment of inertia (m^4^) at Section 1-1, $C_{{L_{1} }}$ = ${\mathrm{m}}L_{1}$ = Vertical subgrade coefficient at Section 1-1.

#### Power series solution

The solution to Eq. (15) is:

18 $$\left[ \begin{gathered} \overline{y(z)} \hfill \\ \overline{\varphi (z)} \hfill \\ \overline{M(z)} \hfill \\ \overline{Q(z)} \hfill \\ \end{gathered} \right] = \left[ \begin{gathered} A_{1} {\kern 1pt} {\kern 1pt} {\kern 1pt} {\kern 1pt} {\kern 1pt} B_{1} {\kern 1pt} {\kern 1pt} {\kern 1pt} {\kern 1pt} {\kern 1pt} C_{1} {\kern 1pt} {\kern 1pt} {\kern 1pt} D_{1} \hfill \\ A_{2} {\kern 1pt} {\kern 1pt} {\kern 1pt} {\kern 1pt} {\kern 1pt} B_{2} {\kern 1pt} {\kern 1pt} {\kern 1pt} {\kern 1pt} {\kern 1pt} C_{2} {\kern 1pt} {\kern 1pt} {\kern 1pt} D_{2} \hfill \\ A_{3} {\kern 1pt} {\kern 1pt} {\kern 1pt} {\kern 1pt} {\kern 1pt} B_{3} {\kern 1pt} {\kern 1pt} {\kern 1pt} {\kern 1pt} {\kern 1pt} C_{3} {\kern 1pt} {\kern 1pt} {\kern 1pt} D_{3} \hfill \\ A_{4} {\kern 1pt} {\kern 1pt} {\kern 1pt} {\kern 1pt} {\kern 1pt} B_{4} {\kern 1pt} {\kern 1pt} {\kern 1pt} {\kern 1pt} {\kern 1pt} C_{4} {\kern 1pt} {\kern 1pt} {\kern 1pt} D_{4} \hfill \\ \end{gathered} \right]{\kern 1pt} {\kern 1pt} {\kern 1pt} {\kern 1pt} \left[ \begin{gathered} \overline{{y_{0} }} \hfill \\ \overline{{\varphi_{0} }} \hfill \\ \overline{{M_{0} }} \hfill \\ \overline{{Q_{0} }} \hfill \\ \end{gathered} \right]$$

Applying dimensionless boundary conditions Eq. (16) yields:

19 $$\left[ \begin{gathered} \overline{{y_{0} }} \hfill \\ \overline{{\varphi_{0} }} \hfill \\ \overline{{\varphi_{1} }} \hfill \\ \end{gathered} \right] = \left[ \begin{gathered} \delta_{{\overline{{{\mathrm{y}}_{0} }} \overline{{M_{0} }} }} {\kern 1pt} {\kern 1pt} {\kern 1pt} {\kern 1pt} {\kern 1pt} \delta_{{\overline{{y_{0} }} \overline{{Q_{0} }} }} {\kern 1pt} {\kern 1pt} {\kern 1pt} {\kern 1pt} \hfill \\ \delta_{{\overline{{\varphi_{0} }} \overline{{M_{0} }} }} {\kern 1pt} {\kern 1pt} {\kern 1pt} {\kern 1pt} {\kern 1pt} \delta_{{\overline{{\varphi_{0} }} \overline{{Q_{0} }} }} {\kern 1pt} {\kern 1pt} {\kern 1pt} \hfill \\ \delta_{{\overline{{\varphi_{1} }} \overline{{M_{0} }} }} {\kern 1pt} {\kern 1pt} {\kern 1pt} {\kern 1pt} {\kern 1pt} \delta_{{\overline{{\varphi_{1} }} \overline{{Q_{0} }} }} {\kern 1pt} {\kern 1pt} {\kern 1pt} {\kern 1pt} \hfill \\ \end{gathered} \right]{\kern 1pt} {\kern 1pt} {\kern 1pt} {\kern 1pt} \left[ \begin{gathered} \overline{{M_{0} }} \hfill \\ \overline{{Q_{0} }} \hfill \\ \end{gathered} \right]$$

with coefficients:

20 $$\left\{ \begin{gathered} \delta_{{\overline{{{\mathrm{y}}_{0} }} \overline{{M_{0} }} }} = \frac{{(B_{11} C_{31} - B_{31} C_{11} ) + K_{{L_{1} }} (B_{11} C_{21} - B_{21} C_{11} )}}{{(A_{11} B_{31} - A_{31} B_{11} ) + K_{{L_{1} }} (A_{11} B_{21} - A_{21} B_{11} )}} \hfill \\ \delta_{{\overline{{y_{0} }} \overline{{Q_{0} }} }} = \frac{{(B_{11} D_{31} - B_{31} D_{11} ) + K_{{L_{1} }} (B_{11} D_{21} - B_{21} D_{11} )}}{{(A_{11} B_{31} - A_{31} B_{11} ) + K_{{L_{1} }} (A_{11} B_{21} - A_{21} B_{11} )}} \hfill \\ \delta_{{\overline{{\varphi_{0} }} \overline{{M_{0} }} }} = \frac{{(A_{31} C_{11} - A_{11} C_{31} ) + K_{{L_{1} }} (A_{21} C_{11} - A_{11} C_{21} )}}{{(A_{11} B_{31} - A_{31} B_{11} ) + K_{{L_{1} }} (A_{11} B_{21} - A_{21} B_{11} )}} \hfill \\ \delta_{{\overline{{\varphi_{0} }} \overline{{Q_{0} }} }} = \frac{{(A_{31} D_{11} - A_{11} D_{31} ) + K_{{L_{1} }} (A_{21} D_{11} - A_{11} D_{21} )}}{{(A_{11} B_{31} - A_{31} B_{11} ) + K_{{L_{1} }} (A_{11} B_{21} - A_{21} B_{11} )}} \hfill \\ \delta_{{\overline{{\varphi_{1} }} \overline{{M_{0} }} }} = \frac{{(A_{31} B_{21} - A_{21} B_{31} )C_{11} + (A_{11} B_{31} - A_{31} B_{11} )C_{21} + (A_{21} B_{11} - A_{11} B_{21} )C_{31} }}{{(A_{11} B_{31} - A_{31} B_{11} ) + K_{{L_{1} }} (A_{11} B_{21} - A_{21} B_{11} )}} \hfill \\ \delta_{{\overline{{\varphi_{1} }} \overline{{Q_{0} }} }} = \frac{{(A_{31} B_{21} - A_{21} B_{31} )D_{11} + (A_{11} B_{31} - A_{31} B_{11} )D_{21} + (A_{21} B_{11} - A_{11} B_{21} )D_{31} }}{{(A_{11} B_{31} - A_{31} B_{11} ) + K_{{L_{1} }} (A_{11} B_{21} - A_{21} B_{11} )}} \hfill \\ \end{gathered} \right.$$

Here, $K_{{L_{1} }} = \frac{{C_{{L_{1} }} I_{{L_{1} }} }}{{\alpha_{1} E_{1} I_{1} }}$,$X_{{{\mathrm{mn}}}}$ denotes coefficient $X_{{\mathrm{m}}}$ at Section *n* (*X* ∈ {A,B,C,D}, *m* ∈ {1,2,3,4}).

Substituting $\overline{{{\mathrm{y}}_{0} }}$ and $\overline{{\varphi_{0} }}$ into Eq. (18) gives the mechanical response.

### Critical state

Rotation $\overline{{\varphi_{1} }}$ at Section 1-1 satisfies:

21 $$\varphi_{1} = \overline{{\varphi_{1} }} = \delta_{{\overline{{\varphi_{1} }} \overline{{M_{0} }} }} \cdot \overline{{M_{0} }} + \delta_{{\overline{{\varphi_{1} }} \overline{{Q_{0} }} }} \cdot \overline{{Q_{0} }}$$

When $\overline{{\varphi_{1} }}$ reaches the limit $\overline{{\varphi^{0} }}$ (governed by joint geometry), the gap development phase terminates. Further loading initiates force transfer to the lower segment. The critical load is:

22 $$\overline{{\varphi_{1} }} = \overline{{\varphi_{0} }} = \delta_{{\overline{{\varphi_{1} }} \overline{{M_{0} }} }} \cdot \overline{{M_{0} }} + \delta_{{\overline{{\varphi_{1} }} \overline{{Q_{0} }} }} \cdot \overline{{Q_{0} }}$$

### Collaborative phase

After exceeding the critical load combination, the lower pile segment initiates displacement under joint mobilization. Both segments now function as an integrated system. Separate governing equations are established for each segment, coupled through mechanical joint behavior.

#### Upper segment solution

The upper segment’s governing equation resembles Eq. (19) but with modified boundary conditions:

23 $$\frac{{d^{4} y}}{{dz^{4} }} + \alpha_{1}^{5} zy = 0$$

where $\alpha_{1}$, ${\mathrm{b}}_{01}$ and $E_{1} I_{1}$ are defined as in Section 3.1.1 Boundary conditions incorporate intermediate variables ${\mathrm{y}}_{1}$ (displacement) and $\varphi_{1}$ (rotation) at Section 1-1:

24 $$\left\{ \begin{gathered} \left\{ \begin{gathered} M({\mathrm{z}} = 0) = 0 \hfill \\ Q(z = 0) = 0 \hfill \\ \end{gathered} \right.,{\text{Free - head }} \hfill \\ \left\{ \begin{gathered} y(z = L_{1} ) = {\mathrm{y}}_{1} \hfill \\ \varphi (z = L_{1} ) = \varphi_{1} \hfill \\ \end{gathered} \right.,Prescribed \, Displacement/Rotation \, at \, Base \hfill \\ \end{gathered} \right.$$

The power series solution (Eq. 25) yields:

25 $$\left[ \begin{gathered} \overline{y(z)} \hfill \\ \overline{\varphi (z)} \hfill \\ \overline{M(z)} \hfill \\ \overline{Q(z)} \hfill \\ \end{gathered} \right] = \left[ \begin{gathered} A_{1} {\kern 1pt} {\kern 1pt} {\kern 1pt} {\kern 1pt} {\kern 1pt} B_{1} {\kern 1pt} {\kern 1pt} {\kern 1pt} {\kern 1pt} {\kern 1pt} {\kern 1pt} C_{1} {\kern 1pt} {\kern 1pt} {\kern 1pt} {\kern 1pt} {\kern 1pt} D_{1} \hfill \\ A_{2} {\kern 1pt} {\kern 1pt} {\kern 1pt} {\kern 1pt} {\kern 1pt} B_{2} {\kern 1pt} {\kern 1pt} {\kern 1pt} {\kern 1pt} {\kern 1pt} C_{2} {\kern 1pt} {\kern 1pt} {\kern 1pt} D_{2} \hfill \\ A_{3} {\kern 1pt} {\kern 1pt} {\kern 1pt} {\kern 1pt} {\kern 1pt} B_{3} {\kern 1pt} {\kern 1pt} {\kern 1pt} {\kern 1pt} {\kern 1pt} C_{3} {\kern 1pt} {\kern 1pt} {\kern 1pt} D_{3} \hfill \\ A_{4} {\kern 1pt} {\kern 1pt} {\kern 1pt} {\kern 1pt} {\kern 1pt} B_{4} {\kern 1pt} {\kern 1pt} {\kern 1pt} {\kern 1pt} {\kern 1pt} C_{4} {\kern 1pt} {\kern 1pt} {\kern 1pt} D_{4} \hfill \\ \end{gathered} \right]{\kern 1pt} {\kern 1pt} {\kern 1pt} {\kern 1pt} \left[ \begin{gathered} \overline{{y_{0} }} \hfill \\ \overline{{\varphi_{0} }} \hfill \\ \overline{{M_{0} }} \hfill \\ \overline{{Q_{0} }} \hfill \\ \end{gathered} \right]$$

Applying dimensionless boundary conditions Eq. (24) yields:

26 $$\left[ \begin{gathered} \overline{{y_{0} }} \hfill \\ \overline{{\varphi_{0} }} \hfill \\ \end{gathered} \right] = \left[ \begin{gathered} \delta_{{\overline{{{\mathrm{y}}_{0} }} \overline{{y_{1} }} }} {\kern 1pt} {\kern 1pt} {\kern 1pt} {\kern 1pt} {\kern 1pt} \delta_{{\overline{{y_{0} }} \overline{{\varphi_{1} }} }} {\kern 1pt} {\kern 1pt} {\kern 1pt} {\kern 1pt} {\kern 1pt} {\kern 1pt} {\kern 1pt} \delta_{{\overline{{{\mathrm{y}}_{0} }} \overline{{M_{0} }} }} {\kern 1pt} {\kern 1pt} {\kern 1pt} {\kern 1pt} {\kern 1pt} \delta_{{\overline{{y_{0} }} \overline{{Q_{0} }} }} \hfill \\ \delta_{{\overline{{\varphi_{0} }} \overline{{y_{1} }} }} {\kern 1pt} {\kern 1pt} {\kern 1pt} {\kern 1pt} {\kern 1pt} \delta_{{\overline{{\varphi_{0} }} \overline{{\varphi_{1} }} }} {\kern 1pt} {\kern 1pt} {\kern 1pt} {\kern 1pt} {\kern 1pt} \delta_{{\overline{{\varphi_{0} }} \overline{{M_{0} }} }} {\kern 1pt} {\kern 1pt} {\kern 1pt} {\kern 1pt} {\kern 1pt} \delta_{{\overline{{\varphi_{0} }} \overline{{Q_{0} }} }} {\kern 1pt} {\kern 1pt} {\kern 1pt} {\kern 1pt} {\kern 1pt} {\kern 1pt} {\kern 1pt} \hfill \\ \end{gathered} \right]{\kern 1pt} {\kern 1pt} {\kern 1pt} {\kern 1pt} \left[ \begin{gathered} \overline{{{\mathrm{y}}_{1} }} \hfill \\ \overline{{\varphi_{1} }} \hfill \\ \overline{{M_{0} }} \hfill \\ \overline{{Q_{0} }} \hfill \\ \end{gathered} \right]$$

with coefficients $\delta$ given in Eq. (27).

27 $${\kern 1pt} {\kern 1pt} \left\{ \begin{gathered} \delta_{{\overline{{{\mathrm{y}}_{0} }} \overline{{y_{1} }} }} = - \frac{{B_{21} }}{{A_{21} B_{11} - A_{11} B_{21} }} \hfill \\ {\kern 1pt} \delta_{{\overline{{y_{0} }} \overline{{\varphi_{1} }} }} = \frac{{B_{11} }}{{A_{21} B_{11} - A_{11} B_{21} }} \hfill \\ \delta_{{\overline{{{\mathrm{y}}_{0} }} \overline{{M_{0} }} }} = \frac{{B_{21} C_{11} - B_{11} C_{21} }}{{A_{21} B_{11} - A_{11} B_{21} }} \hfill \\ \delta_{{\overline{{y_{0} }} \overline{{Q_{0} }} }} = \frac{{B_{21} D_{11} - B_{11} D_{21} }}{{A_{21} B_{11} - A_{11} B_{21} }} \hfill \\ \delta_{{\overline{{\varphi_{0} }} \overline{{y_{1} }} }} = \frac{{A_{21} }}{{A_{21} B_{11} - A_{11} B_{21} }} \hfill \\ \delta_{{\overline{{\varphi_{0} }} \overline{{\varphi_{1} }} }} = - \frac{{A_{11} }}{{A_{21} B_{11} - A_{11} B_{21} }} \hfill \\ \delta_{{\overline{{\varphi_{0} }} \overline{{M_{0} }} }} = - \frac{{A_{21} C_{11} - A_{11} C_{21} }}{{A_{21} B_{11} - A_{11} B_{21} }} \hfill \\ \delta_{{\overline{{\varphi_{0} }} \overline{{Q_{0} }} }} = - \frac{{A_{21} D_{11} - A_{11} D_{21} }}{{A_{21} B_{11} - A_{11} B_{21} }} \hfill \\ \end{gathered} \right.$$

Substituting into Eq. (25) expresses the solution in terms of $\overline{{{\mathrm{y}}_{1} }}$ and $\overline{{\varphi_{1} }}$:

28 $$\left[ \begin{gathered} \overline{y(z)} \hfill \\ \overline{\varphi (z)} \hfill \\ \overline{M(z)} \hfill \\ \overline{Q(z)} \hfill \\ \end{gathered} \right] = \left[ {\begin{array}{*{20}c} {\delta_{{\overline{{\mathrm{y}}} \overline{{y_{1} }} }} } & {\delta_{{\overline{y} \overline{{\varphi_{1} }} }} } & {\delta_{{\overline{{\mathrm{y}}} \overline{{M_{0} }} }} } & {\delta_{{\overline{y} \overline{{Q_{0} }} }} } \\ {\delta_{{\overline{\varphi } \overline{{y_{1} }} }} } & {\delta_{{\overline{\varphi } \overline{{\varphi_{1} }} }} } & {\delta_{{\overline{\varphi } \overline{{M_{0} }} }} } & {\delta_{{\overline{\varphi } \overline{{Q_{0} }} }} } \\ {\delta_{{\overline{M} \overline{{y_{1} }} }} } & {\delta_{{\overline{M} \overline{{\varphi_{1} }} }} } & {\delta_{{\overline{M} \overline{{M_{0} }} }} } & {\delta_{{\overline{M} \overline{{Q_{0} }} }} } \\ {\delta_{{\overline{Q} \overline{{y_{1} }} }} } & {\delta_{{\overline{Q} \overline{{\varphi_{1} }} }} } & {\delta_{{\overline{Q} \overline{{M_{0} }} }} } & {\delta_{{\overline{Q} \overline{{Q_{0} }} }} } \\ \end{array} } \right]\;\left[ \begin{gathered} \overline{{{\mathrm{y}}_{1} }} \hfill \\ \overline{{\varphi_{1} }} \hfill \\ \overline{{M_{0} }} \hfill \\ \overline{{Q_{0} }} \hfill \\ \end{gathered} \right]\quad \;(0 \le L \le L_{1} )$$

Coefficients $\delta$ (Eq. 29) quantify boundary-condition influences at any depth ${\mathrm{L}}$ (via $\alpha {\mathrm{L}}$).

29 $$\left\{ \begin{gathered} \delta_{{\overline{{\mathrm{y}}} \overline{{{\mathrm{y}}_{1} }} }} = \frac{{B_{1} A_{21} - A_{1} B_{21} }}{{A_{21} B_{11} - A_{11} B_{21} }} \hfill \\ \delta_{{\overline{y} \overline{{\varphi_{1} }} }} = \frac{{A_{1} B_{11} - B_{1} A_{11} }}{{A_{21} B_{11} - A_{11} B_{21} }} \hfill \\ \delta_{{\overline{y} \overline{{M_{0} }} }} = \frac{{A_{1} (B_{21} C_{11} - B_{11} C_{21} ) - B_{1} (A_{21} C_{11} - A_{11} C_{21} )}}{{A_{21} B_{11} - A_{11} B_{21} }} + C_{1} \hfill \\ \delta_{{\overline{y} \overline{{Q_{0} }} }} = \frac{{A_{1} (B_{21} D_{11} - B_{11} D_{21} ) - B_{1} (A_{21} D_{11} - A_{11} D_{21} )}}{{A_{21} B_{11} - A_{11} B_{21} }} + D_{1} \hfill \\ \delta_{{\overline{\varphi } \overline{{{\mathrm{y}}_{1} }} }} = \frac{{B_{2} A_{21} - A_{2} B_{21} }}{{A_{21} B_{11} - A_{11} B_{21} }} \hfill \\ \delta_{{\overline{\varphi } \overline{{\varphi_{1} }} }} = \frac{{A_{2} B_{11} - B_{2} A_{11} }}{{A_{21} B_{11} - A_{11} B_{21} }} \hfill \\ \delta_{{\overline{\varphi } \overline{{M_{0} }} }} = \frac{{A_{2} (B_{21} C_{11} - B_{11} C_{21} ) - B_{2} (A_{21} C_{11} - A_{11} C_{21} )}}{{A_{21} B_{11} - A_{11} B_{21} }} + C_{2} \hfill \\ \delta_{{\overline{\varphi } \overline{{Q_{0} }} }} = \frac{{A_{2} (B_{21} D_{11} - B_{11} D_{21} ) - B_{2} (A_{21} D_{11} - A_{11} D_{21} )}}{{A_{21} B_{11} - A_{11} B_{21} }} + D_{2} \hfill \\ \delta_{{\overline{M} \overline{{{\mathrm{y}}_{1} }} }} = \frac{{B_{3} A_{21} - A_{3} B_{21} }}{{A_{21} B_{11} - A_{11} B_{21} }} \hfill \\ \delta_{{\overline{M} \overline{{\varphi_{1} }} }} = \frac{{A_{3} B_{11} - B_{3} A_{11} }}{{A_{21} B_{11} - A_{11} B_{21} }} \hfill \\ \delta_{{\overline{M} \overline{{M_{0} }} }} = \frac{{A_{3} (B_{21} C_{11} - B_{11} C_{21} ) - B_{3} (A_{21} C_{11} - A_{11} C_{21} )}}{{A_{21} B_{11} - A_{11} B_{21} }} + C_{3} \hfill \\ \delta_{{\overline{M} \overline{{Q_{0} }} }} = \frac{{A_{3} (B_{21} D_{11} - B_{11} D_{21} ) - B_{3} (A_{21} D_{11} - A_{11} D_{21} )}}{{A_{21} B_{11} - A_{11} B_{21} }} + D_{3} \hfill \\ \delta_{{\overline{Q} \overline{{{\mathrm{y}}_{1} }} }} = \frac{{B_{4} A_{21} - A_{4} B_{21} }}{{A_{21} B_{11} - A_{11} B_{21} }} \hfill \\ \delta_{{\overline{Q} \overline{{\varphi_{1} }} }} = \frac{{A_{4} B_{11} - B_{4} A_{11} }}{{A_{21} B_{11} - A_{11} B_{21} }} \hfill \\ \delta_{{\overline{Q} \overline{{M_{0} }} }} = \frac{{A_{4} (B_{21} C_{11} - B_{11} C_{21} ) - B_{4} (A_{21} C_{11} - A_{11} C_{21} )}}{{A_{21} B_{11} - A_{11} B_{21} }} + C_{4} \hfill \\ \delta_{{\overline{Q} \overline{{Q_{0} }} }} = \frac{{A_{4} (B_{21} D_{11} - B_{11} D_{21} ) - B_{4} (A_{21} D_{11} - A_{11} D_{21} )}}{{A_{21} B_{11} - A_{11} B_{21} }} + D_{4} \hfill \\ \end{gathered} \right.$$

#### Lower segment solution via fictitious pile method

**Fig. 5:**
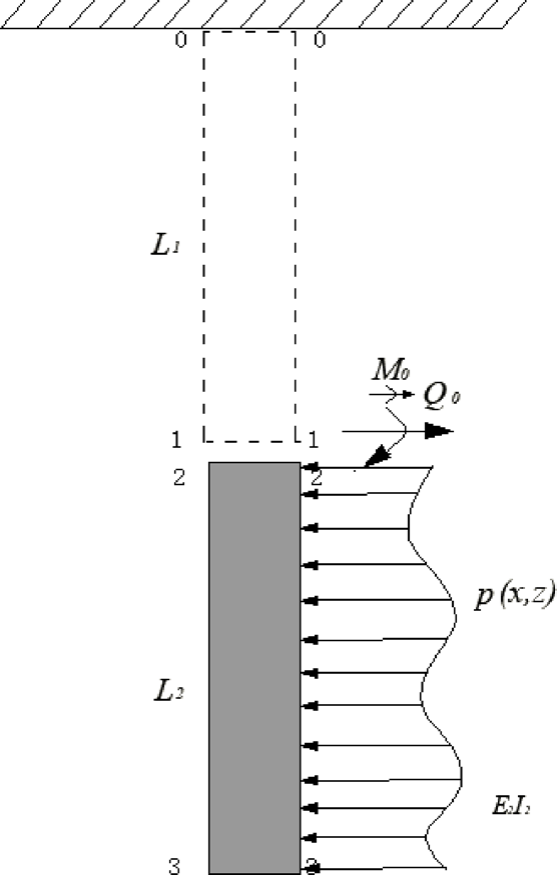
Conceptual illustration of the Fictitious Pile Method for analyzing segmented piles.

A fictitious full-length pile (length $L_{1} + L_{2}$) is conceptualized (Fig. 5), with its lower portion ( $L_{1} \le z \le L_{1} + L_{2}$ ) matching the actual lower segment when deformations at Section 2-2 are identical.

Governing equation:

30 $$\frac{{d^{4} y}}{{dz^{4} }} + \alpha_{2}^{5} zy = 0$$

Parameters $\alpha_{2}$, ${\mathrm{b}}_{02}$, $E_{2} I_{2}$ follow Section 3.1.1 definitions. Boundary conditions:

- Free tip: $$\left\{ \begin{gathered} M^{\Delta } ({\mathrm{z}} = L_{1} + L_{2} ) = 0 \hfill \\ Q^{\Delta } ({\mathrm{z}} = L_{1} + L_{2} ) = 0 \hfill \\ \end{gathered} \right.$$
- Section 2-2: $$\left\{ \begin{gathered} {\mathrm{y}}^{\Delta } ({\mathrm{z}} = L_{1} ) = y_{2}^{\Delta } \hfill \\ \varphi^{\Delta } ({\mathrm{z}} = L_{1} ) = \varphi_{2}^{\Delta } \hfill \\ \end{gathered} \right.$$

Since the full-length pile is an equivalent fictitious model, the parameters ${\mathrm{y}}^{\Delta }$, $\varphi^{\Delta }$,${\mathrm{M}}^{\Delta }$ and ${\mathrm{Q}}^{\Delta }$ do not physically exist. Thus, the superscript $\Delta$ is used to distinguish these fictitious quantities.

The dimensionless solution takes the form:

31 $$\left[ \begin{gathered} \overline{y(z)}^{\Delta } \hfill \\ \overline{\varphi (z)}^{\Delta } \hfill \\ \overline{M(z)}^{\Delta } \hfill \\ \overline{Q(z)}^{\Delta } \hfill \\ \end{gathered} \right] = \left[ \begin{gathered} A_{1} {\kern 1pt} {\kern 1pt} {\kern 1pt} {\kern 1pt} {\kern 1pt} B_{1} {\kern 1pt} {\kern 1pt} {\kern 1pt} {\kern 1pt} {\kern 1pt} {\kern 1pt} C_{1} {\kern 1pt} {\kern 1pt} {\kern 1pt} {\kern 1pt} {\kern 1pt} D_{1} \hfill \\ A_{2} {\kern 1pt} {\kern 1pt} {\kern 1pt} {\kern 1pt} {\kern 1pt} B_{2} {\kern 1pt} {\kern 1pt} {\kern 1pt} {\kern 1pt} {\kern 1pt} C_{2} {\kern 1pt} {\kern 1pt} {\kern 1pt} D_{2} \hfill \\ A_{3} {\kern 1pt} {\kern 1pt} {\kern 1pt} {\kern 1pt} {\kern 1pt} B_{3} {\kern 1pt} {\kern 1pt} {\kern 1pt} {\kern 1pt} {\kern 1pt} C_{3} {\kern 1pt} {\kern 1pt} {\kern 1pt} D_{3} \hfill \\ A_{4} {\kern 1pt} {\kern 1pt} {\kern 1pt} {\kern 1pt} {\kern 1pt} B_{4} {\kern 1pt} {\kern 1pt} {\kern 1pt} {\kern 1pt} {\kern 1pt} C_{4} {\kern 1pt} {\kern 1pt} {\kern 1pt} D_{4} \hfill \\ \end{gathered} \right]{\kern 1pt} {\kern 1pt} {\kern 1pt} {\kern 1pt} \left[ \begin{gathered} \overline{{y_{0} }}^{\Delta } \hfill \\ \overline{{\varphi_{0} }}^{\Delta } \hfill \\ \overline{{M_{0} }}^{\Delta } \hfill \\ \overline{{Q_{0} }}^{\Delta } \hfill \\ \end{gathered} \right]$$

1. Apply tip conditions to express $\overline{y}_{0}^{\Delta }$,$\overline{\varphi }_{0}^{\Delta }$:

32 $$\left[ \begin{gathered} \overline{{y_{0} }}^{\Delta } \hfill \\ \overline{{\varphi_{0} }}^{\Delta } \hfill \\ \end{gathered} \right] = \left[ \begin{gathered} \delta_{{\overline{{{\mathrm{y}}_{0} }}^{\Delta } \overline{{M_{0} }}^{\Delta } }} {\kern 1pt} {\kern 1pt} {\kern 1pt} {\kern 1pt} {\kern 1pt} \delta_{{\overline{{y_{0} }}^{\Delta } \overline{{Q_{0} }}^{\Delta } }} \hfill \\ \delta_{{\overline{{\varphi_{0} }}^{\Delta } \overline{{M_{0}^{\Delta } }} }} {\kern 1pt} {\kern 1pt} {\kern 1pt} {\kern 1pt} {\kern 1pt} {\kern 1pt} {\kern 1pt} {\kern 1pt} {\kern 1pt} {\kern 1pt} {\kern 1pt} \delta_{{\overline{{\varphi_{0} }}^{\Delta } \overline{{Q_{0} }}^{\Delta } }} {\kern 1pt} {\kern 1pt} {\kern 1pt} {\kern 1pt} {\kern 1pt} {\kern 1pt} {\kern 1pt} \hfill \\ \end{gathered} \right]{\kern 1pt} {\kern 1pt} {\kern 1pt} {\kern 1pt} \left[ \begin{gathered} \overline{{M_{0} }}^{\Delta } \hfill \\ \overline{{Q_{0} }}^{\Delta } \hfill \\ \end{gathered} \right]$$

Coefficients $\delta$ computed via Eq. (33).

33 $${\kern 1pt} {\kern 1pt} \left\{ \begin{gathered} \delta_{{\overline{{{\mathrm{y}}_{0} }}^{\Delta } \overline{{M_{0} }}^{\Delta } }} = \frac{{B_{43} C_{33} - B_{33} C_{43} }}{{A_{43} B_{33} - A_{33} B_{43} }} \hfill \\ \delta_{{\overline{{y_{0} }}^{\Delta } \overline{{Q_{0} }}^{\Delta } }} = \frac{{B_{43} D_{33} - B_{33} D_{43} }}{{A_{43} B_{33} - A_{33} B_{43} }} \hfill \\ \delta_{{\overline{{\varphi_{0} }}^{\Delta } \overline{{M_{0} }}^{\Delta } }} = - \frac{{A_{43} C_{33} - A_{33} C_{43} }}{{A_{43} B_{33} - A_{33} B_{43} }} \hfill \\ \delta_{{\overline{{\varphi_{0} }}^{\Delta } \overline{{Q_{0} }}^{\Delta } }} = - \frac{{A_{43} D_{33} - A_{33} D_{43} }}{{A_{43} B_{33} - A_{33} B_{43} }} \hfill \\ \end{gathered} \right.$$

(2) Substitute into (31):

34 $$\left[ \begin{gathered} \overline{y(z)}^{\Delta } \hfill \\ \overline{\varphi (z)}^{\Delta } \hfill \\ \overline{M(z)}^{\Delta } \hfill \\ \overline{Q(z)}^{\Delta } \hfill \\ \end{gathered} \right] = \left[ \begin{gathered} \delta_{{\overline{{\mathrm{y}}}^{\Delta } \overline{{M_{0} }}^{\Delta } }} {\kern 1pt} {\kern 1pt} {\kern 1pt} {\kern 1pt} {\kern 1pt} {\kern 1pt} {\kern 1pt} {\kern 1pt} {\kern 1pt} {\kern 1pt} {\kern 1pt} {\kern 1pt} {\kern 1pt} \delta_{{\overline{y}^{\Delta } \overline{{Q_{0} }}^{\Delta } }} \hfill \\ {\kern 1pt} \delta_{{\overline{\varphi }^{\Delta } \overline{{M_{0} }}^{\Delta } }} {\kern 1pt} {\kern 1pt} {\kern 1pt} {\kern 1pt} {\kern 1pt} {\kern 1pt} {\kern 1pt} {\kern 1pt} {\kern 1pt} {\kern 1pt} {\kern 1pt} {\kern 1pt} \delta_{{\overline{\varphi }^{\Delta } \overline{{Q_{0} }}^{\Delta } }} {\kern 1pt} {\kern 1pt} \hfill \\ {\kern 1pt} \delta_{{\overline{M}^{\Delta } \overline{{M_{0} }}^{\Delta } }} {\kern 1pt} {\kern 1pt} {\kern 1pt} {\kern 1pt} {\kern 1pt} {\kern 1pt} {\kern 1pt} {\kern 1pt} {\kern 1pt} \delta_{{\overline{M}^{\Delta } \overline{{Q_{0} }}^{\Delta } }} {\kern 1pt} \hfill \\ {\kern 1pt} \delta_{{\overline{Q}^{\Delta } \overline{{M_{0} }}^{\Delta } }} {\kern 1pt} {\kern 1pt} {\kern 1pt} {\kern 1pt} {\kern 1pt} {\kern 1pt} {\kern 1pt} {\kern 1pt} {\kern 1pt} {\kern 1pt} {\kern 1pt} \delta_{{\overline{Q}^{\Delta } \overline{{Q_{0} }}^{\Delta } }} {\kern 1pt} \hfill \\ \end{gathered} \right]{\kern 1pt} {\kern 1pt} {\kern 1pt} {\kern 1pt} \left[ \begin{gathered} \overline{{M_{0} }}^{\Delta } \hfill \\ \overline{{Q_{0} }}^{\Delta } \hfill \\ \end{gathered} \right]$$

Coefficients $\delta$ defined in Eq. (35).

35 $$\left[ \begin{gathered} \delta_{{\overline{{\mathrm{y}}}^{\Delta } \overline{{M_{0} }}^{\Delta } }} {\kern 1pt} {\kern 1pt} {\kern 1pt} {\kern 1pt} {\kern 1pt} {\kern 1pt} {\kern 1pt} {\kern 1pt} {\kern 1pt} {\kern 1pt} \delta_{{\overline{y}^{\Delta } \overline{{Q_{0} }}^{\Delta } }} {\kern 1pt} \hfill \\ \delta_{{\overline{\varphi }^{\Delta } \overline{{M_{0} }}^{\Delta } }} {\kern 1pt} {\kern 1pt} {\kern 1pt} {\kern 1pt} {\kern 1pt} {\kern 1pt} {\kern 1pt} {\kern 1pt} {\kern 1pt} \delta_{{\overline{\varphi }^{\Delta } \overline{{Q_{0} }}^{\Delta } }} {\kern 1pt} \hfill \\ \delta_{{\overline{M}^{\Delta } \overline{{M_{0} }}^{\Delta } {\kern 1pt} {\kern 1pt} {\kern 1pt} {\kern 1pt} {\kern 1pt} }} {\kern 1pt} \delta_{{\overline{M}^{\Delta } \overline{{Q_{0} }}^{\Delta } }} \hfill \\ \delta_{{\overline{Q}^{\Delta } \overline{{M_{0} }}^{\Delta } }} {\kern 1pt} {\kern 1pt} {\kern 1pt} {\kern 1pt} {\kern 1pt} {\kern 1pt} {\kern 1pt} {\kern 1pt} \delta_{{\overline{Q}^{\Delta } \overline{{Q_{0} }}^{\Delta } }} \hfill \\ \end{gathered} \right] = \left[ \begin{gathered} A_{1} {\kern 1pt} {\kern 1pt} {\kern 1pt} {\kern 1pt} {\kern 1pt} {\kern 1pt} B_{1} \hfill \\ A_{2} {\kern 1pt} {\kern 1pt} {\kern 1pt} {\kern 1pt} {\kern 1pt} {\kern 1pt} B_{2} \hfill \\ A_{3} {\kern 1pt} {\kern 1pt} {\kern 1pt} {\kern 1pt} {\kern 1pt} {\kern 1pt} B_{3} \hfill \\ A_{4} {\kern 1pt} {\kern 1pt} {\kern 1pt} {\kern 1pt} {\kern 1pt} {\kern 1pt} B_{4} \hfill \\ \end{gathered} \right]\left[ \begin{gathered} \delta_{{\overline{{{\mathrm{y}}_{0} }}^{\Delta } \overline{{M_{0} }}^{\Delta } }} {\kern 1pt} {\kern 1pt} {\kern 1pt} {\kern 1pt} {\kern 1pt} {\kern 1pt} {\kern 1pt} \delta_{{\overline{{{\mathrm{y}}_{0} }}^{\Delta } \overline{{Q_{0} }}^{\Delta } }} \hfill \\ {\kern 1pt} \delta_{{\overline{{\varphi_{0} }}^{\Delta } \overline{{M_{0} }}^{\Delta } }} {\kern 1pt} {\kern 1pt} {\kern 1pt} {\kern 1pt} \delta_{{\overline{{\varphi_{0} }}^{\Delta } \overline{{Q_{0} }}^{\Delta } }} {\kern 1pt} {\kern 1pt} {\kern 1pt} \hfill \\ \end{gathered} \right]{\kern 1pt} {\kern 1pt} {\kern 1pt} {\kern 1pt} + {\kern 1pt} {\kern 1pt} {\kern 1pt} \left[ \begin{gathered} C_{1} {\kern 1pt} {\kern 1pt} {\kern 1pt} {\kern 1pt} {\kern 1pt} {\kern 1pt} D_{1} \hfill \\ C_{2} {\kern 1pt} {\kern 1pt} {\kern 1pt} {\kern 1pt} {\kern 1pt} {\kern 1pt} D_{2} \hfill \\ C_{3} {\kern 1pt} {\kern 1pt} {\kern 1pt} {\kern 1pt} {\kern 1pt} {\kern 1pt} D_{2} \hfill \\ C_{4} {\kern 1pt} {\kern 1pt} {\kern 1pt} {\kern 1pt} {\kern 1pt} {\kern 1pt} D_{4} \hfill \\ \end{gathered} \right]$$

(3) Enforce Section 2-2 conditions to solve for $\overline{M}_{0}^{\Delta }$, $\overline{Q}_{0}^{\Delta }$:

36 $$\left[ \begin{gathered} \overline{{M_{0} }}^{\Delta } \hfill \\ \overline{{Q_{0} }}^{\Delta } \hfill \\ \end{gathered} \right] = \left[ \begin{gathered} \delta_{{\overline{{{\mathrm{M}}_{0} }}^{\Delta } \overline{{{\mathrm{y}}_{2} }}^{\Delta } }} {\kern 1pt} {\kern 1pt} {\kern 1pt} {\kern 1pt} {\kern 1pt} \delta_{{\overline{{M_{0} }}^{\Delta } \overline{{\varphi_{2} }}^{\Delta } }} \hfill \\ \delta_{{\overline{{Q_{0} }}^{\Delta } \overline{{y_{2}^{\Delta } }} }} {\kern 1pt} {\kern 1pt} {\kern 1pt} {\kern 1pt} {\kern 1pt} {\kern 1pt} {\kern 1pt} {\kern 1pt} {\kern 1pt} {\kern 1pt} {\kern 1pt} \delta_{{\overline{{Q_{0} }}^{\Delta } \overline{{\varphi_{2} }}^{\Delta } }} {\kern 1pt} {\kern 1pt} {\kern 1pt} {\kern 1pt} {\kern 1pt} {\kern 1pt} {\kern 1pt} \hfill \\ \end{gathered} \right]{\kern 1pt} {\kern 1pt} {\kern 1pt} {\kern 1pt} \left[ \begin{gathered} \overline{{y_{2} }}^{\Delta } \hfill \\ \overline{{\varphi_{2} }}^{\Delta } \hfill \\ \end{gathered} \right]$$

Coefficients $\delta$ from Eq. (37).

37 $${\kern 1pt} {\kern 1pt} \left\{ \begin{gathered} \delta_{{\overline{{{\mathrm{M}}_{0} }}^{\Delta } \overline{{{\mathrm{y}}_{2} }}^{\Delta } }} = \frac{{\delta_{{\overline{{\varphi_{2} }}^{\Delta } \overline{{Q_{0} }}^{\Delta } }} }}{{\delta_{{\overline{{{\mathrm{y}}_{2} }}^{\Delta } \overline{{M_{0} }}^{\Delta } }} \cdot \delta_{{\overline{{\varphi_{2} }}^{\Delta } \overline{{Q_{0} }}^{\Delta } }} - \delta_{{\overline{{\varphi_{2} }}^{\Delta } \overline{{M_{0} }}^{\Delta } }} \cdot \delta_{{\overline{{y_{2} }}^{\Delta } \overline{{Q_{0} }}^{\Delta } }} }} \hfill \\ \delta_{{\overline{{M_{0} }}^{\Delta } \overline{{\varphi_{2} }}^{\Delta } }} = \frac{{ - \delta_{{\overline{{y_{2} }}^{\Delta } \overline{{Q_{0} }}^{\Delta } }} }}{{\delta_{{\overline{{{\mathrm{y}}_{2} }}^{\Delta } \overline{{M_{0} }}^{\Delta } }} \cdot \delta_{{\overline{{\varphi_{2} }}^{\Delta } \overline{{Q_{0} }}^{\Delta } }} - \delta_{{\overline{{\varphi_{2} }}^{\Delta } \overline{{M_{0} }}^{\Delta } }} \cdot \delta_{{\overline{{y_{2} }}^{\Delta } \overline{{Q_{0} }}^{\Delta } }} }} \hfill \\ \delta_{{\overline{{Q_{0} }}^{\Delta } \overline{{y_{2}^{\Delta } }} }} = \frac{{\delta_{{\overline{{y_{2} }}^{\Delta } \overline{{M_{0} }}^{\Delta } }} }}{{\delta_{{\overline{{{\mathrm{y}}_{2} }}^{\Delta } \overline{{M_{0} }}^{\Delta } }} \cdot \delta_{{\overline{{\varphi_{2} }}^{\Delta } \overline{{Q_{0} }}^{\Delta } }} - \delta_{{\overline{{\varphi_{2} }}^{\Delta } \overline{{M_{0} }}^{\Delta } }} \cdot \delta_{{\overline{{y_{2} }}^{\Delta } \overline{{Q_{0} }}^{\Delta } }} }} \hfill \\ {\kern 1pt} {\kern 1pt} \delta_{{\overline{{Q_{0} }}^{\Delta } \overline{{\varphi_{2} }}^{\Delta } }} = \frac{{ - \delta_{{\overline{{\varphi_{2} }}^{\Delta } \overline{{M_{0} }}^{\Delta } }} }}{{\delta_{{\overline{{{\mathrm{y}}_{2} }}^{\Delta } \overline{{M_{0} }}^{\Delta } }} \cdot \delta_{{\overline{{\varphi_{2} }}^{\Delta } \overline{{Q_{0} }}^{\Delta } }} - \delta_{{\overline{{\varphi_{2} }}^{\Delta } \overline{{M_{0} }}^{\Delta } }} \cdot \delta_{{\overline{{y_{2} }}^{\Delta } \overline{{Q_{0} }}^{\Delta } }} }} \hfill \\ \end{gathered} \right.$$

(4) Final solution for fictitious pile:

38 $$\left[ \begin{gathered} \overline{y(z)}^{\Delta } \hfill \\ \overline{\varphi (z)}^{\Delta } \hfill \\ \overline{M(z)}^{\Delta } \hfill \\ \overline{Q(z)}^{\Delta } \hfill \\ \end{gathered} \right] = \left[ \begin{gathered} \delta_{{\overline{{\mathrm{y}}}^{\Delta } \overline{{{\mathrm{y}}_{2} }}^{\Delta } }} {\kern 1pt} {\kern 1pt} {\kern 1pt} {\kern 1pt} {\kern 1pt} {\kern 1pt} {\kern 1pt} {\kern 1pt} {\kern 1pt} {\kern 1pt} {\kern 1pt} {\kern 1pt} {\kern 1pt} \delta_{{\overline{y}^{\Delta } \overline{{\varphi_{2} }}^{\Delta } }} \hfill \\ {\kern 1pt} \delta_{{\overline{\varphi }^{\Delta } \overline{{y_{2} }}^{\Delta } }} {\kern 1pt} {\kern 1pt} {\kern 1pt} {\kern 1pt} {\kern 1pt} {\kern 1pt} {\kern 1pt} {\kern 1pt} {\kern 1pt} {\kern 1pt} {\kern 1pt} {\kern 1pt} \delta_{{\overline{\varphi }^{\Delta } \overline{{\varphi_{2} }}^{\Delta } }} {\kern 1pt} {\kern 1pt} \hfill \\ {\kern 1pt} \delta_{{\overline{M}^{\Delta } \overline{{y_{2} }}^{\Delta } }} {\kern 1pt} {\kern 1pt} {\kern 1pt} {\kern 1pt} {\kern 1pt} {\kern 1pt} {\kern 1pt} {\kern 1pt} {\kern 1pt} \delta_{{\overline{M}^{\Delta } \overline{{\varphi_{2} }}^{\Delta } }} {\kern 1pt} \hfill \\ {\kern 1pt} \delta_{{\overline{Q}^{\Delta } \overline{{y_{2} }}^{\Delta } }} {\kern 1pt} {\kern 1pt} {\kern 1pt} {\kern 1pt} {\kern 1pt} {\kern 1pt} {\kern 1pt} {\kern 1pt} {\kern 1pt} {\kern 1pt} {\kern 1pt} \delta_{{\overline{Q}^{\Delta } \overline{{\varphi_{2} }}^{\Delta } }} {\kern 1pt} \hfill \\ \end{gathered} \right]{\kern 1pt} {\kern 1pt} {\kern 1pt} {\kern 1pt} \left[ \begin{gathered} \overline{{y_{2} }}^{\Delta } \hfill \\ \overline{{\varphi_{2} }}^{\Delta } \hfill \\ \end{gathered} \right]$$

Coefficients $\delta$ in Eq. (39).

39 $${\kern 1pt} {\kern 1pt} \left\{ \begin{gathered} \delta _{{\overline{{\mathrm{y}}} ^{\Delta } \overline{{{\mathrm{y}}_{2} }} ^{\Delta } }} = \frac{{\delta _{{\overline{y} ^{\Delta } \overline{{M_{0} }} ^{\Delta } }} \cdot \delta _{{\overline{{\varphi _{2} }} ^{\Delta } \overline{{Q_{0} }} ^{\Delta } }} - \delta _{{\overline{y} ^{\Delta } \overline{{Q_{0} }} ^{\Delta } }} \cdot \delta _{{\overline{{\varphi _{2} }} ^{\Delta } \overline{{M_{0} }} ^{\Delta } }} }}{{\delta _{{\overline{{{\mathrm{y}}_{2} }} ^{\Delta } \overline{{M_{0} }} ^{\Delta } }} \cdot \delta _{{\overline{{\varphi _{2} }} ^{\Delta } \overline{{Q_{0} }} ^{\Delta } }} - \delta _{{\overline{{\varphi _{2} }} ^{\Delta } \overline{{M_{0} }} ^{\Delta } }} \cdot \delta _{{\overline{{y_{2} }} ^{\Delta } \overline{{Q_{0} }} ^{\Delta } }} }} \hfill \\ \delta _{{\overline{y} ^{\Delta } \overline{{\varphi _{2} }} ^{\Delta } }} = \frac{{\delta _{{\overline{y} ^{\Delta } \overline{{Q_{0} }} ^{\Delta } }} \cdot \delta _{{\overline{{y_{2} }} ^{\Delta } \overline{{M_{0} }} ^{\Delta } }} - \delta _{{\overline{y} ^{\Delta } \overline{{M_{0} }} ^{\Delta } }} \cdot \delta _{{\overline{{y_{2} }} ^{\Delta } \overline{{Q_{0} }} ^{\Delta } }} }}{{\delta _{{\overline{{{\mathrm{y}}_{2} }} ^{\Delta } \overline{{M_{0} }} ^{\Delta } }} \cdot \delta _{{\overline{{\varphi _{2} }} ^{\Delta } \overline{{Q_{0} }} ^{\Delta } }} - \delta _{{\overline{{\varphi _{2} }} ^{\Delta } \overline{{M_{0} }} ^{\Delta } }} \cdot \delta _{{\overline{{y_{2} }} ^{\Delta } \overline{{Q_{0} }} ^{\Delta } }} }} \hfill \\ \delta _{{\overline{\varphi } ^{\Delta } \overline{{y_{2} ^{\Delta } }} }} = \frac{{\delta _{{\overline{\varphi } ^{\Delta } \overline{{M_{0} }} ^{\Delta } }} \cdot \delta _{{\overline{{\varphi _{2} }} ^{\Delta } \overline{{Q_{0} }} ^{\Delta } }} - \delta _{{\overline{\varphi } ^{\Delta } \overline{{Q_{0} }} ^{\Delta } }} \cdot \delta _{{\overline{{\varphi _{2} }} ^{\Delta } \overline{{M_{0} }} ^{\Delta } }} }}{{\delta _{{\overline{{{\mathrm{y}}_{2} }} ^{\Delta } \overline{{M_{0} }} ^{\Delta } }} \cdot \delta _{{\overline{{\varphi _{2} }} ^{\Delta } \overline{{Q_{0} }} ^{\Delta } }} - \delta _{{\overline{{\varphi _{2} }} ^{\Delta } \overline{{M_{0} }} ^{\Delta } }} \cdot \delta _{{\overline{{y_{2} }} ^{\Delta } \overline{{Q_{0} }} ^{\Delta } }} }} \hfill \\ {\kern 1pt} {\kern 1pt} \delta _{{\overline{\varphi } ^{\Delta } \overline{{\varphi _{2} }} ^{\Delta } }} = \frac{{\delta _{{\overline{\varphi } ^{\Delta } \overline{{Q_{0} }} ^{\Delta } }} \cdot \delta _{{\overline{{y_{2} }} ^{\Delta } \overline{{M_{0} }} ^{\Delta } }} - \delta _{{\overline{\varphi } ^{\Delta } \overline{{M_{0} }} ^{\Delta } }} \cdot \delta _{{\overline{{y_{2} }} ^{\Delta } \overline{{Q_{0} }} ^{\Delta } }} }}{{\delta _{{\overline{{{\mathrm{y}}_{2} }} ^{\Delta } \overline{{M_{0} }} ^{\Delta } }} \cdot \delta _{{\overline{{\varphi _{2} }} ^{\Delta } \overline{{Q_{0} }} ^{\Delta } }} - \delta _{{\overline{{\varphi _{2} }} ^{\Delta } \overline{{M_{0} }} ^{\Delta } }} \cdot \delta _{{\overline{{y_{2} }} ^{\Delta } \overline{{Q_{0} }} ^{\Delta } }} }} \hfill \\ \delta _{{\overline{M} ^{\Delta } \overline{{{\mathrm{y}}_{2} }} ^{\Delta } }} = \frac{{\delta _{{\overline{M} ^{\Delta } \overline{{M_{0} }} ^{\Delta } }} \cdot \delta _{{\overline{{\varphi _{2} }} ^{\Delta } \overline{{Q_{0} }} ^{\Delta } }} - \delta _{{\overline{M} ^{\Delta } \overline{{Q_{0} }} ^{\Delta } }} \cdot \delta _{{\overline{{\varphi _{2} }} ^{\Delta } \overline{{M_{0} }} ^{\Delta } }} }}{{\delta _{{\overline{{{\mathrm{y}}_{2} }} ^{\Delta } \overline{{M_{0} }} ^{\Delta } }} \cdot \delta _{{\overline{{\varphi _{2} }} ^{\Delta } \overline{{Q_{0} }} ^{\Delta } }} - \delta _{{\overline{{\varphi _{2} }} ^{\Delta } \overline{{M_{0} }} ^{\Delta } }} \cdot \delta _{{\overline{{y_{2} }} ^{\Delta } \overline{{Q_{0} }} ^{\Delta } }} }} \hfill \\ \delta _{{\overline{M} ^{\Delta } \overline{{\varphi _{2} }} ^{\Delta } }} = \frac{{\delta _{{\overline{M} ^{\Delta } \overline{{Q_{0} }} ^{\Delta } }} \cdot \delta _{{\overline{{y_{2} }} ^{\Delta } \overline{{M_{0} }} ^{\Delta } }} - \delta _{{\overline{M} ^{\Delta } \overline{{M_{0} }} ^{\Delta } }} \cdot \delta _{{\overline{{y_{2} }} ^{\Delta } \overline{{Q_{0} }} ^{\Delta } }} }}{{\delta _{{\overline{{{\mathrm{y}}_{2} }} ^{\Delta } \overline{{M_{0} }} ^{\Delta } }} \cdot \delta _{{\overline{{\varphi _{2} }} ^{\Delta } \overline{{Q_{0} }} ^{\Delta } }} - \delta _{{\overline{{\varphi _{2} }} ^{\Delta } \overline{{M_{0} }} ^{\Delta } }} \cdot \delta _{{\overline{{y_{2} }} ^{\Delta } \overline{{Q_{0} }} ^{\Delta } }} }} \hfill \\ \delta _{{\overline{Q} ^{\Delta } \overline{{y_{2} ^{\Delta } }} }} = \frac{{\delta _{{\overline{Q} ^{\Delta } \overline{{M_{0} }} ^{\Delta } }} \cdot \delta _{{\overline{{\varphi _{2} }} ^{\Delta } \overline{{Q_{0} }} ^{\Delta } }} - \delta _{{\overline{Q} ^{\Delta } \overline{{Q_{0} }} ^{\Delta } }} \cdot \delta _{{\overline{{\varphi _{2} }} ^{\Delta } \overline{{M_{0} }} ^{\Delta } }} }}{{\delta _{{\overline{{{\mathrm{y}}_{2} }} ^{\Delta } \overline{{M_{0} }} ^{\Delta } }} \cdot \delta _{{\overline{{\varphi _{2} }} ^{\Delta } \overline{{Q_{0} }} ^{\Delta } }} - \delta _{{\overline{{\varphi _{2} }} ^{\Delta } \overline{{M_{0} }} ^{\Delta } }} \cdot \delta _{{\overline{{y_{2} }} ^{\Delta } \overline{{Q_{0} }} ^{\Delta } }} }} \hfill \\ {\kern 1pt} {\kern 1pt} \delta _{{\overline{Q} ^{\Delta } \overline{{\varphi _{2} }} ^{\Delta } }} = \frac{{\delta _{{\overline{Q} ^{\Delta } \overline{{Q_{0} }} ^{\Delta } }} \cdot \delta _{{\overline{{y_{2} }} ^{\Delta } \overline{{M_{0} }} ^{\Delta } }} - \delta _{{\overline{Q} ^{\Delta } \overline{{M_{0} }} ^{\Delta } }} \cdot \delta _{{\overline{{y_{2} }} ^{\Delta } \overline{{Q_{0} }} ^{\Delta } }} }}{{\delta _{{\overline{{{\mathrm{y}}_{2} }} ^{\Delta } \overline{{M_{0} }} ^{\Delta } }} \cdot \delta _{{\overline{{\varphi _{2} }} ^{\Delta } \overline{{Q_{0} }} ^{\Delta } }} - \delta _{{\overline{{\varphi _{2} }} ^{\Delta } \overline{{M_{0} }} ^{\Delta } }} \cdot \delta _{{\overline{{y_{2} }} ^{\Delta } \overline{{Q_{0} }} ^{\Delta } }} }} \hfill \\ \end{gathered} \right.$$

For ${\mathrm{L}}_{1} \le {\mathrm{L}} \le {\mathrm{L}}_{1} + {\mathrm{L}}_{2}$, the fictitious pile’s solution equals the actual lower segment’s response, ultimately expressing displacements/forces in terms of $\overline{y}_{2}$($\overline{y}_{2} = \overline{y}_{2}^{\Delta }$)and $\overline{\varphi }_{2}$($\overline{\varphi }_{2} = \overline{\varphi }_{2}^{\Delta }$) in Eq. 40.

40 $$\left[ \begin{gathered} \overline{y(z)} \hfill \\ \overline{\varphi (z)} \hfill \\ \overline{M(z)} \hfill \\ \overline{Q(z)} \hfill \\ \end{gathered} \right] = \left[ {\begin{array}{*{20}c} {\delta_{{\overline{{\mathrm{y}}}^{\Delta } \overline{{{\mathrm{y}}_{2} }}^{\Delta } }} } & {\delta_{{\overline{y}^{\Delta } \overline{{\varphi_{2} }}^{\Delta } }} } \\ {\delta_{{\overline{\varphi }^{\Delta } \overline{{y_{2} }}^{\Delta } }} } & {\delta_{{\overline{\varphi }^{\Delta } \overline{{\varphi_{2} }}^{\Delta } }} } \\ {\delta_{{\overline{M}^{\Delta } \overline{{y_{2} }}^{\Delta } }} } & {\delta_{{\overline{M}^{\Delta } \overline{{\varphi_{2} }}^{\Delta } }} } \\ {\delta_{{\overline{Q}^{\Delta } \overline{{y_{2} }}^{\Delta } }} } & {\delta_{{\overline{Q}^{\Delta } \overline{{\varphi_{2} }}^{\Delta } }} } \\ \end{array} } \right]\;\left[ \begin{gathered} \overline{{y_{2} }} \hfill \\ \overline{{\varphi_{2} }} \hfill \\ \end{gathered} \right]\quad \;(L_{1} \le L \le L_{1} + L_{2} )$$

#### Integrated pile response

For the mechanically-jointed pile under top loads $M_{0}$,$Q_{0}$:

28 $$\left[ \begin{gathered} \overline{y(z)} \hfill \\ \overline{\varphi (z)} \hfill \\ \overline{M(z)} \hfill \\ \overline{Q(z)} \hfill \\ \end{gathered} \right] = \left[ \begin{gathered} \delta_{{\overline{{\mathrm{y}}} \overline{{y_{1} }} }} {\kern 1pt} {\kern 1pt} {\kern 1pt} {\kern 1pt} {\kern 1pt} {\kern 1pt} {\kern 1pt} {\kern 1pt} {\kern 1pt} {\kern 1pt} \delta_{{\overline{y} \overline{{\varphi_{1} }} }} {\kern 1pt} {\kern 1pt} {\kern 1pt} {\kern 1pt} {\kern 1pt} {\kern 1pt} {\kern 1pt} \delta_{{\overline{{\mathrm{y}}} \overline{{M_{0} }} }} {\kern 1pt} {\kern 1pt} {\kern 1pt} {\kern 1pt} {\kern 1pt} {\kern 1pt} {\kern 1pt} {\kern 1pt} {\kern 1pt} \delta_{{\overline{y} \overline{{Q_{0} }} }} \hfill \\ \delta_{{\overline{\varphi } \overline{{y_{1} }} }} {\kern 1pt} {\kern 1pt} {\kern 1pt} {\kern 1pt} {\kern 1pt} {\kern 1pt} {\kern 1pt} {\kern 1pt} \delta_{{\overline{\varphi } \overline{{\varphi_{1} }} }} {\kern 1pt} {\kern 1pt} {\kern 1pt} {\kern 1pt} {\kern 1pt} {\kern 1pt} {\kern 1pt} {\kern 1pt} {\kern 1pt} \delta_{{\overline{\varphi } \overline{{M_{0} }} }} {\kern 1pt} {\kern 1pt} {\kern 1pt} {\kern 1pt} {\kern 1pt} {\kern 1pt} {\kern 1pt} {\kern 1pt} \delta_{{\overline{\varphi } \overline{{Q_{0} }} }} {\kern 1pt} {\kern 1pt} \hfill \\ \delta_{{\overline{M} \overline{{y_{1} }} }} {\kern 1pt} {\kern 1pt} {\kern 1pt} {\kern 1pt} {\kern 1pt} \delta_{{\overline{M} \overline{{\varphi_{1} }} }} {\kern 1pt} {\kern 1pt} {\kern 1pt} {\kern 1pt} {\kern 1pt} \delta_{{\overline{M} \overline{{M_{0} }} }} {\kern 1pt} {\kern 1pt} {\kern 1pt} {\kern 1pt} {\kern 1pt} \delta_{{\overline{M} \overline{{Q_{0} }} }} {\kern 1pt} \hfill \\ {\kern 1pt} \delta_{{\overline{Q} \overline{{y_{1} }} }} {\kern 1pt} {\kern 1pt} {\kern 1pt} {\kern 1pt} {\kern 1pt} {\kern 1pt} \delta_{{\overline{Q} \overline{{\varphi_{1} }} }} {\kern 1pt} {\kern 1pt} {\kern 1pt} {\kern 1pt} {\kern 1pt} {\kern 1pt} {\kern 1pt} {\kern 1pt} \delta_{{\overline{Q} \overline{{M_{0} }} }} {\kern 1pt} {\kern 1pt} {\kern 1pt} {\kern 1pt} {\kern 1pt} {\kern 1pt} {\kern 1pt} \delta_{{\overline{Q} \overline{{Q_{0} }} }} {\kern 1pt} \hfill \\ \end{gathered} \right]{\kern 1pt} {\kern 1pt} {\kern 1pt} {\kern 1pt} \left[ \begin{gathered} \overline{{{\mathrm{y}}_{1} }} \hfill \\ \overline{{\varphi_{1} }} \hfill \\ \overline{{M_{0} }} \hfill \\ \overline{{Q_{0} }} \hfill \\ \end{gathered} \right]\quad (0 \le L \le L_{1} )$$

40 $$\left[ \begin{gathered} \overline{y(z)} \hfill \\ \overline{\varphi (z)} \hfill \\ \overline{M(z)} \hfill \\ \overline{Q(z)} \hfill \\ \end{gathered} \right] = \left[ {\begin{array}{*{20}c} {\delta_{{\overline{{\mathrm{y}}}^{\Delta } \overline{{{\mathrm{y}}_{2} }}^{\Delta } }} } & {\delta_{{\overline{y}^{\Delta } \overline{{\varphi_{2} }}^{\Delta } }} } \\ {\delta_{{\overline{\varphi }^{\Delta } \overline{{y_{2} }}^{\Delta } }} } & {\delta_{{\overline{\varphi }^{\Delta } \overline{{\varphi_{2} }}^{\Delta } }} } \\ {\delta_{{\overline{M}^{\Delta } \overline{{y_{2} }}^{\Delta } }} } & {\delta_{{\overline{M}^{\Delta } \overline{{\varphi_{2} }}^{\Delta } }} } \\ {\delta_{{\overline{Q}^{\Delta } \overline{{y_{2} }}^{\Delta } }} } & {\delta_{{\overline{Q}^{\Delta } \overline{{\varphi_{2} }}^{\Delta } }} } \\ \end{array} } \right]\;\left[ \begin{gathered} \overline{{y_{2} }} \hfill \\ \overline{{\varphi_{2} }} \hfill \\ \end{gathered} \right]\;\quad \left( {L_{1} \le L \le L_{1} + L_{2} } \right)$$

Unknowns $\overline{y}_{1}$,$\overline{\varphi }_{1}$,$\overline{y}_{2}$,$\overline{\varphi }_{2}$ are resolved through:

1. Geometric Compatibility (rigid joint):

41 $$\left\{ \begin{gathered} \overline{{{\mathrm{y}}_{1} }} = \overline{{{\mathrm{y}}_{2} }} \hfill \\ \overline{{\varphi_{1} }} + \overline{{\varphi^{0} }} = \overline{{\varphi_{2} }} \hfill \\ \end{gathered} \right.$$

(2) Force Equilibrium (full force transfer):

42 $$\left\{ \begin{gathered} \overline{{{\mathrm{M}}_{1} }} = \overline{{{\mathrm{M}}_{2} }} \hfill \\ \overline{{Q_{1} }} = \overline{{Q_{2} }} \hfill \\ \end{gathered} \right.$$

Solving Eqs.(28),(40)–(42) eliminates intermediate variables (Eqs.43–44), yielding the complete pile response.

43 $$\left[ \begin{gathered} \overline{{y_{1} }} \hfill \\ \overline{{\varphi_{1} }} \hfill \\ \end{gathered} \right] = \left[ \begin{gathered} \delta_{{\overline{{\mathrm{y}}}_{1} \overline{{\varphi^{0} }} }} {\kern 1pt} {\kern 1pt} {\kern 1pt} {\kern 1pt} {\kern 1pt} {\kern 1pt} {\kern 1pt} {\kern 1pt} {\kern 1pt} {\kern 1pt} {\kern 1pt} {\kern 1pt} {\kern 1pt} \delta_{{\overline{{y_{1} }} \overline{{M_{0} }} }} {\kern 1pt} {\kern 1pt} {\kern 1pt} {\kern 1pt} {\kern 1pt} {\kern 1pt} {\kern 1pt} {\kern 1pt} {\kern 1pt} \delta_{{\overline{{y_{1} }} \overline{{Q_{0} }} }} \hfill \\ {\kern 1pt} \delta_{{\overline{\varphi }_{1} \overline{{\varphi^{0} }} }} {\kern 1pt} {\kern 1pt} {\kern 1pt} {\kern 1pt} {\kern 1pt} {\kern 1pt} {\kern 1pt} {\kern 1pt} {\kern 1pt} {\kern 1pt} {\kern 1pt} {\kern 1pt} {\kern 1pt} \delta_{{\overline{{\varphi_{1} }} \overline{{M_{0} }} }} {\kern 1pt} {\kern 1pt} {\kern 1pt} {\kern 1pt} {\kern 1pt} {\kern 1pt} {\kern 1pt} {\kern 1pt} {\kern 1pt} \delta_{{\overline{{\varphi_{1} }} \overline{{Q_{0} }} }} {\kern 1pt} {\kern 1pt} \hfill \\ \end{gathered} \right]{\kern 1pt} {\kern 1pt} {\kern 1pt} {\kern 1pt} \left[ \begin{gathered} \overline{{\varphi^{0} }} \hfill \\ \overline{{M_{0} }} \hfill \\ \overline{{Q_{0} }} \hfill \\ \end{gathered} \right]$$

Coefficients $\delta$ in Eq. (44).

44 $${\kern 1pt} {\kern 1pt} \left\{ \begin{gathered} \delta _{{\overline{{{\mathrm{y}}_{1} }} \overline{{\varphi ^{0} }} }} = \frac{{(\delta _{{\overline{{Q_{1} }} \overline{{\varphi _{1} }} }} - \delta _{{\overline{{Q_{2} }} ^{\Delta } \overline{{\varphi _{2} }} ^{\Delta } }} ) \cdot \delta _{{\overline{{M_{2} }} ^{\Delta } \overline{{\varphi _{2} }} ^{\Delta } }} - (\delta _{{\overline{{M_{1} }} \overline{{\varphi _{1} }} }} - \delta _{{\overline{{M_{2} }} ^{\Delta } \overline{{\varphi _{2} }} ^{\Delta } }} ) \cdot \delta _{{\overline{{Q_{2} }} ^{\Delta } \overline{{\varphi _{2} }} ^{\Delta } }} }}{{(\delta _{{\overline{{M_{1} }} \overline{{{\mathrm{y}}_{1} }} }} - \delta _{{\overline{{M_{2} }} ^{\Delta } \overline{{y_{2} }} ^{\Delta } }} ) \cdot (\delta _{{\overline{{Q_{1} }} \overline{{\varphi _{1} }} }} - \delta _{{\overline{{Q_{2} }} ^{\Delta } \overline{{\varphi _{2} }} ^{\Delta } }} ) - (\delta _{{\overline{{Q_{1} }} \overline{{{\mathrm{y}}_{1} }} }} - \delta _{{\overline{{Q_{2} }} ^{\Delta } \overline{{y_{2} }} ^{\Delta } }} ) \cdot (\delta _{{\overline{{M_{1} }} \overline{{\varphi _{1} }} }} - \delta _{{\overline{{M_{2} }} ^{\Delta } \overline{{\varphi _{2} }} ^{\Delta } }} )}} \hfill \\ \delta _{{\overline{{{\mathrm{y}}_{1} }} \overline{{M_{0} }} }} = \frac{{ - (\delta _{{\overline{{Q_{1} }} \overline{{\varphi _{1} }} }} - \delta _{{\overline{{Q_{2} }} ^{\Delta } \overline{{\varphi _{2} }} ^{\Delta } }} ) \cdot \delta _{{\overline{{M_{1} }} \overline{{M_{0} }} }} + (\delta _{{\overline{{M_{1} }} \overline{{\varphi _{1} }} }} - \delta _{{\overline{{M_{2} }} ^{\Delta } \overline{{\varphi _{2} }} ^{\Delta } }} ) \cdot \delta _{{\overline{{Q_{1} }} \overline{{M_{0} }} }} }}{{(\delta _{{\overline{{M_{1} }} \overline{{{\mathrm{y}}_{1} }} }} - \delta _{{\overline{{M_{2} }} ^{\Delta } \overline{{y_{2} }} ^{\Delta } }} ) \cdot (\delta _{{\overline{{Q_{1} }} \overline{{\varphi _{1} }} }} - \delta _{{\overline{{Q_{2} }} ^{\Delta } \overline{{\varphi _{2} }} ^{\Delta } }} ) - (\delta _{{\overline{{Q_{1} }} \overline{{{\mathrm{y}}_{1} }} }} - \delta _{{\overline{{Q_{2} }} ^{\Delta } \overline{{y_{2} }} ^{\Delta } }} ) \cdot (\delta _{{\overline{{M_{1} }} \overline{{\varphi _{1} }} }} - \delta _{{\overline{{M_{2} }} ^{\Delta } \overline{{\varphi _{2} }} ^{\Delta } }} )}} \hfill \\ \delta _{{\overline{{{\mathrm{y}}_{1} }} \overline{{Q_{0} }} }} = \frac{{ - (\delta _{{\overline{{Q_{1} }} \overline{{\varphi _{1} }} }} - \delta _{{\overline{{Q_{2} }} ^{\Delta } \overline{{\varphi _{2} }} ^{\Delta } }} ) \cdot \delta _{{\overline{{M_{1} }} \overline{{Q_{0} }} }} + (\delta _{{\overline{{M_{1} }} \overline{{\varphi _{1} }} }} - \delta _{{\overline{{M_{2} }} ^{\Delta } \overline{{\varphi _{2} }} ^{\Delta } }} ) \cdot \delta _{{\overline{{Q_{1} }} \overline{{Q_{0} }} }} }}{{(\delta _{{\overline{{M_{1} }} \overline{{{\mathrm{y}}_{1} }} }} - \delta _{{\overline{{M_{2} }} ^{\Delta } \overline{{y_{2} }} ^{\Delta } }} ) \cdot (\delta _{{\overline{{Q_{1} }} \overline{{\varphi _{1} }} }} - \delta _{{\overline{{Q_{2} }} ^{\Delta } \overline{{\varphi _{2} }} ^{\Delta } }} ) - (\delta _{{\overline{{Q_{1} }} \overline{{{\mathrm{y}}_{1} }} }} - \delta _{{\overline{{Q_{2} }} ^{\Delta } \overline{{y_{2} }} ^{\Delta } }} ) \cdot (\delta _{{\overline{{M_{1} }} \overline{{\varphi _{1} }} }} - \delta _{{\overline{{M_{2} }} ^{\Delta } \overline{{\varphi _{2} }} ^{\Delta } }} )}} \hfill \\ \delta _{{\overline{{\varphi _{1} }} \overline{{\varphi ^{0} }} }} = \frac{{ - (\delta _{{\overline{{Q_{1} }} \overline{{y_{1} }} }} - \delta _{{\overline{{Q_{2} }} ^{\Delta } \overline{{y_{2} }} ^{\Delta } }} ) \cdot \delta _{{\overline{{M_{2} }} ^{\Delta } \overline{{\varphi _{2} }} ^{\Delta } }} + (\delta _{{\overline{{M_{1} }} \overline{{y_{1} }} }} - \delta _{{\overline{{M_{2} }} ^{\Delta } \overline{{y_{2} }} ^{\Delta } }} ) \cdot \delta _{{\overline{{Q_{2} }} ^{\Delta } \overline{{\varphi _{2} }} ^{\Delta } }} }}{{(\delta _{{\overline{{M_{1} }} \overline{{{\mathrm{y}}_{1} }} }} - \delta _{{\overline{{M_{2} }} ^{\Delta } \overline{{y_{2} }} ^{\Delta } }} ) \cdot (\delta _{{\overline{{Q_{1} }} \overline{{\varphi _{1} }} }} - \delta _{{\overline{{Q_{2} }} ^{\Delta } \overline{{\varphi _{2} }} ^{\Delta } }} ) - (\delta _{{\overline{{Q_{1} }} \overline{{{\mathrm{y}}_{1} }} }} - \delta _{{\overline{{Q_{2} }} ^{\Delta } \overline{{y_{2} }} ^{\Delta } }} ) \cdot (\delta _{{\overline{{M_{1} }} \overline{{\varphi _{1} }} }} - \delta _{{\overline{{M_{2} }} ^{\Delta } \overline{{\varphi _{2} }} ^{\Delta } }} )}} \hfill \\ \delta _{{\overline{{\varphi _{1} }} \overline{{M_{0} }} }} = \frac{{(\delta _{{\overline{{Q_{1} }} \overline{{y_{1} }} }} - \delta _{{\overline{{Q_{2} }} ^{\Delta } \overline{{y_{2} }} ^{\Delta } }} ) \cdot \delta _{{\overline{{M_{1} }} \overline{{M_{0} }} }} - (\delta _{{\overline{{M_{1} }} \overline{{\varphi _{1} }} }} - \delta _{{\overline{{M_{2} }} ^{\Delta } \overline{{\varphi _{2} }} ^{\Delta } }} ) \cdot \delta _{{\overline{{Q_{1} }} \overline{{M_{0} }} }} }}{{(\delta _{{\overline{{M_{1} }} \overline{{{\mathrm{y}}_{1} }} }} - \delta _{{\overline{{M_{2} }} ^{\Delta } \overline{{y_{2} }} ^{\Delta } }} ) \cdot (\delta _{{\overline{{Q_{1} }} \overline{{\varphi _{1} }} }} - \delta _{{\overline{{Q_{2} }} ^{\Delta } \overline{{\varphi _{2} }} ^{\Delta } }} ) - (\delta _{{\overline{{Q_{1} }} \overline{{{\mathrm{y}}_{1} }} }} - \delta _{{\overline{{Q_{2} }} ^{\Delta } \overline{{y_{2} }} ^{\Delta } }} ) \cdot (\delta _{{\overline{{M_{1} }} \overline{{\varphi _{1} }} }} - \delta _{{\overline{{M_{2} }} ^{\Delta } \overline{{\varphi _{2} }} ^{\Delta } }} )}} \hfill \\ \delta _{{\overline{{\varphi _{1} }} \overline{{Q_{0} }} }} = \frac{{(\delta _{{\overline{{Q_{1} }} \overline{{\varphi _{1} }} }} - \delta _{{\overline{{Q_{2} }} ^{\Delta } \overline{{y_{2} }} ^{\Delta } }} ) \cdot \delta _{{\overline{{M_{1} }} \overline{{Q_{0} }} }} - (\delta _{{\overline{{M_{1} }} \overline{{\varphi _{1} }} }} - \delta _{{\overline{{M_{2} }} ^{\Delta } \overline{{\varphi _{2} }} ^{\Delta } }} ) \cdot \delta _{{\overline{{Q_{1} }} \overline{{Q_{0} }} }} }}{{(\delta _{{\overline{{M_{1} }} \overline{{{\mathrm{y}}_{1} }} }} - \delta _{{\overline{{M_{2} }} ^{\Delta } \overline{{y_{2} }} ^{\Delta } }} ) \cdot (\delta _{{\overline{{Q_{1} }} \overline{{\varphi _{1} }} }} - \delta _{{\overline{{Q_{2} }} ^{\Delta } \overline{{\varphi _{2} }} ^{\Delta } }} ) - (\delta _{{\overline{{Q_{1} }} \overline{{{\mathrm{y}}_{1} }} }} - \delta _{{\overline{{Q_{2} }} ^{\Delta } \overline{{y_{2} }} ^{\Delta } }} ) \cdot (\delta _{{\overline{{M_{1} }} \overline{{\varphi _{1} }} }} - \delta _{{\overline{{M_{2} }} ^{\Delta } \overline{{\varphi _{2} }} ^{\Delta } }} )}} \hfill \\ \end{gathered} \right.$$

Substituting $\overline{{{\mathrm{y}}_{1} }}$ and $\overline{{\varphi_{1} }}$ into the geometric compatibility conditions (Eq. 41) yields the intermediate variables $\overline{{{\mathrm{y}}_{2} }}$ and $\overline{{\varphi_{2} }}$ at Section 2-2. The displacement and internal force responses of both upper and lower segments are then obtained by incorporating these intermediate variables into their respective power series solutions. This constitutes the complete pile response formulation for the free-ended mechanically-jointed pile.

## Numerical simulation for validation and parametric investigation

The theoretical model derived in Section 3 offers an efficient analytical tool. To verify its correctness and to examine the mechanical response under more generalized conditions, a parallel numerical modeling study is conducted using the finite element method. The primary objectives of this numerical simulation are twofold: (1) to validate the accuracy of the m-method-based solutions by comparing key response parameters, and (2) to leverage the flexibility of FEM to analyze the influence of factors that are challenging to incorporate analytically, such as the detailed constitutive behavior of the soil and the joint. The model setup and parameters are described below.

### Test scheme and basic parameters

Based on the horizontal static load test of single piles in Reference^30^, a test scheme for conventional and mechanically-jointed piles under free-boundary conditions was established. The *m*-method was employed to calculate mechanical responses for both pile types, comparing the mechanical behavior of mechanically-jointed piles. A numerical model of mechanically-jointed piles was then developed, and the validity of the *m*-method theory was verified by comparing its results with numerical simulations.

Simulation parameters for the pile and reinforcement are adopted from literature^30^. The connector is modeled as a hinge joint with limited rotation, set to $\varphi^{0} = 1000 \times 10^{ - 5} {\mathrm{r}}$. A unidirectional horizontal load is applied. Specific parameters are as follows:

1. Soil Parameters

The soil is simplified as a homogeneous single layer for efficient theoretical calculation and model validation. Its physical and mechanical parameters are listed in Table 1.

(2) Pile Parameters

(2) Pile Parameters

C30 concrete was used. The upper segment of the mechanically-jointed pile was $L_{1} = 3m$ long, and the lower segment was $L_{1} = 15m$ long, with the pile top flush with the ground. The maximum rotation at the connector was $1000 \times 10^{ - 5} {\mathrm{r}}$. According to Reference^24^, the reinforcement scheme was assumed as:

- Main Bars: 9 HRB400 steel bars (Ø25 mm), full-length distribution.
- Stirrups: HPB235 steel bars (Ø12 mm @ 200 mm), 50 mm cover thickness.

Mechanical parameters are listed in Table 2.

**Table 2:** Pile and reinforcement physical and mechanical parameters.

Material	Density ρ (kg/m^3^)	Elastic Modulus E (GPa)	Poisson’s Ratio ν	Diameter d (mm)	Remarks
Concrete pile	2400	30	0.2	600	C30 concrete
Main bars	7850	200	0.3	25	9 bars, uniformly distributed
Stirrups	7850	200	0.3	12	@200 mm, 50 mm cover

(3) Connector Parameters

A Connector Element in ABAQUS simulated the mechanical joint. Hinge behavior was defined via connection properties and constraints (Table 3).

**Table 3:** Connector mechanical parameters.

Connection type	Free DOF	Constrained DOF	Behavior	Rotation limit
Hinge	UR_1_	U_1_, U_2_, U_3_, UR_2_, UR_3_	Limit Stop	0.01 rad

### Model establishment

1. Component Design
  Soil: Cylinder (Ø30 m × 38 m height), diameter = 50 × pile diameter, height = 2.1 × pile length.
  Pile Socket: Central socket (Ø0.6 m × 18 m depth).
2. Meshing
  Element Types:
    Soil & Pile: C3D8R (8-node linear reduced-integration hexahedral elements)
    Reinforcement: T3D2 (2-node truss elements)
  Mesh Sizes:

**Table:** 

Component	Concrete	Reinforcement	Soil
Size	100 mm	200 mm	400 mm

(3) Loading

A horizontal load was applied at the center of the pile top (+ X direction). Coupling Constraints converted the concentrated load into a distributed surface load to avoid stress concentration.

(4) Model Visualization.

**Fig. 6:**
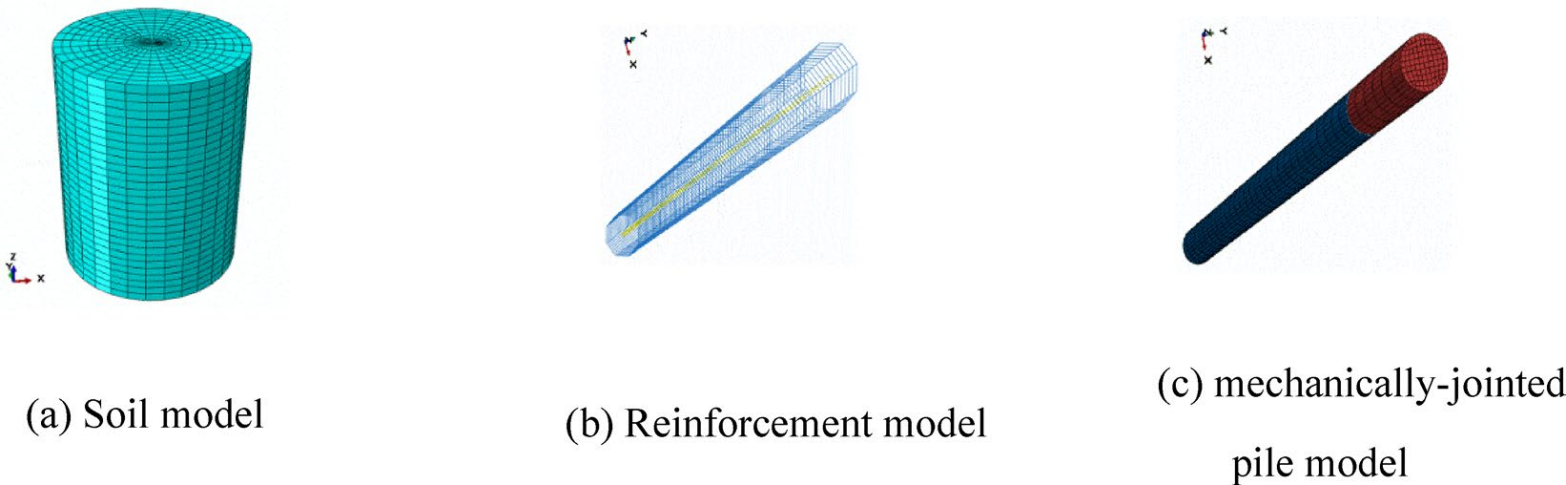
Finite element model used for numerical validation.

The final finite element model is shown in Fig. 6.

### Static analysis

1. Boundary Conditions.

- Soil Side Surfaces: Normal displacement constraints (U₁ = U₂ = 0)
- Soil Bottom Surface: Fully fixed (U₁ = U₂ = U₃ = UR₁ = UR₂ = UR₃ = 0)

(2) Initial Geostatic Equilibrium

Geostatic equilibrium was performed to eliminate initial displacements caused by soil self-weight before applying gravity loads.

(3) Load Steps

- Apply gravity load
- Apply horizontal load at pile top

**Fig. 7:**
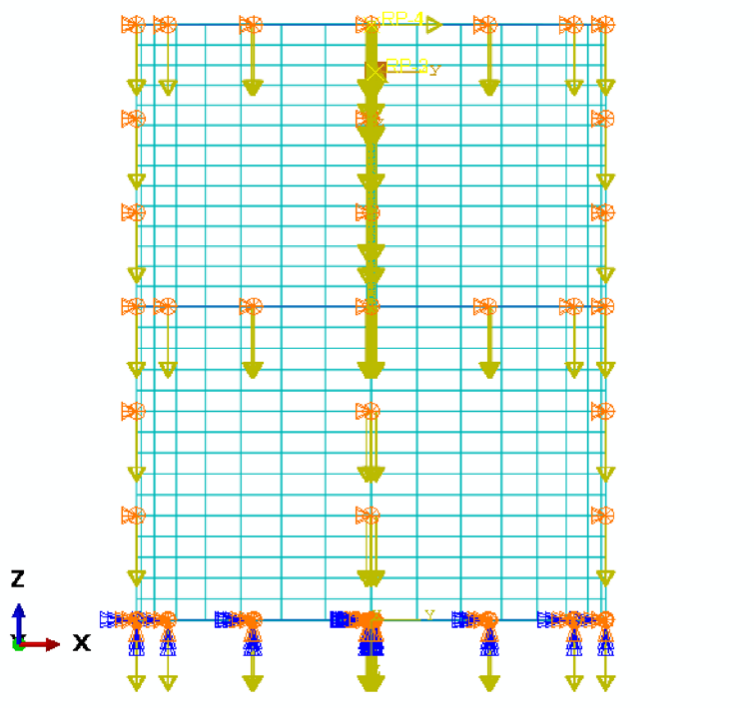
Boundary conditions and loading configuration of the validation model.

Boundary conditions and loading are illustrated in Fig. 7.

(4) Result Extraction Methods

Section Rotation ($\varphi$): Paths were created along symmetric nodes on both sides of the pile surface. The rotation angle $\varphi$ for each section was calculated based on the coordinates of the corresponding nodes before and after deformation.

Internal Force Analysis: The Free-Body Slice Method was used. The entire pile was divided into 37 cross-sections at 0.5 m intervals to extract the bending moment and shear force.

## Analysis of calculation results

### Validation of numerical simulation feasibility

To ensure the comparability between the *m*-method theory and numerical simulation results and the engineering applicability of the model, the numerical simulation results(The horizontal load is 150 kN) were compared with the measured data from Reference^24^. The comparison is presented in Table 4.

**Table 4:** Comparison of calculated pile head displacement and rotation.

Calculation Method	Pile Head Horizontal Displacement (mm)	Error vs. Measured (%)	Pile Head Rotation (× 10⁻^5^ rad)	Error vs. Measured (%)
Numerical Simulation	5.23	3.25	347.4	9.56
Field Measurement	5.06	0	314.2	0

The maximum pile head horizontal displacements obtained from field measurement and numerical simulation are 5.06 mm and 5.23 mm, respectively, with a relative error of 3.25%. The maximum pile head rotations are 314.2 × 10⁻^5^ rad and 347.4 × 10⁻^5^ rad, respectively, with a relative error of 9.56%. Both errors are within acceptable limits, indicating good agreement between the numerical simulation and experimental data. This confirms the reliability of the established pile-soil numerical model for subsequent verification and analysis of the *m*-method theory.

### Verification of *m*-method correctness

#### Analysis of models

**Fig. 8:**
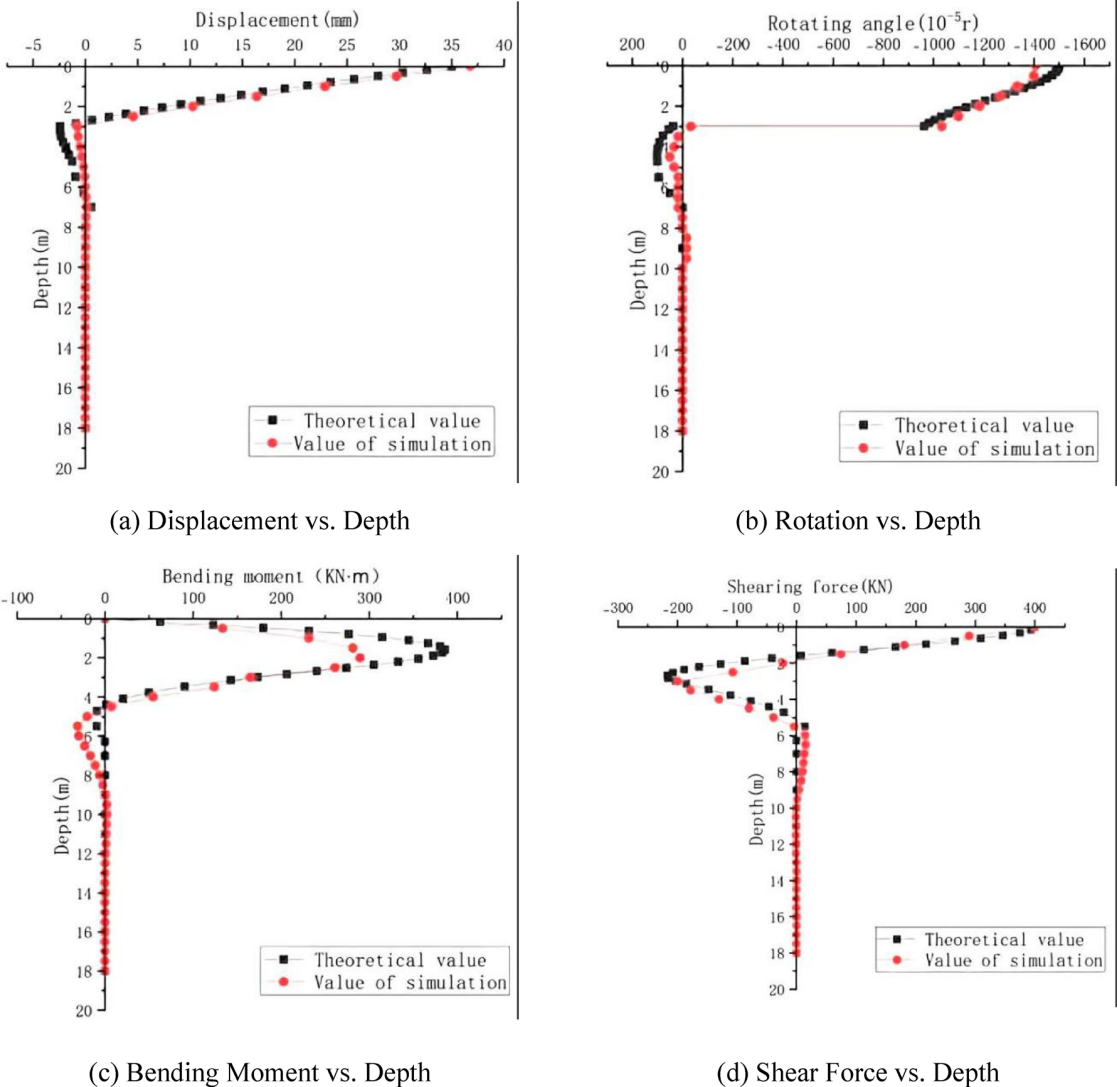
Validation of the extended *m*-method against numerical simulation for key response parameters of the mechanically-jointed pile.

The comparison curves of the displacement and internal force responses along the pile shaft for the mechanically-jointed pile, obtained based on the *m*-method theory and numerical simulation(The horizontal load is 400 kN) , are shown in Fig. 8.

Analysis of Fig. 8 reveals:

1. Displacement: The pile displacement curves calculated by the *m*-method and numerical simulation agree well. The maximum values occur at the pile head, with theoretical (*m*-method) and simulated values of 35.01 mm and 36.78 mm (error 4.8%). The displacement at the connector is negative in both cases (theoretical: − 2.44 mm; simulated: − 0.77 mm).
2. Rotation: The maximum rotation occurs at the pile head. The theoretical (*m*-method) value is − 1499.81 × 10⁻^5^ rad, and the simulated value is − 1406.84 × 10⁻^5^ rad (error 6.2%).
3. Bending Moment: The maximum positive bending moment occurs within 1–3 m below the pile head. The theoretical maximum (385.40 kN m) is at 1.571 m depth, while the simulated maximum (289.60 kN·m) is at 2.0 m depth. The simulated value is smaller, with an error of 24.9%. The maximum negative moment occurs within 4–6 m below the pile head. The theoretical and simulated points of contra-flexure are at 4.398 m and 4.5 m, respectively, which are close.
4. Shear Force: The maximum positive shear force (400 kN) occurs at the pile head. The theoretical maximum negative shear force (− 216.88 kN) is at 2.670 m depth, and the simulated value (− 199.04 kN) is at 3.0 m depth (error 8.2%). The theoretical and simulated depths for zero shear force are 1.571 m and 1.8 m, respectively.

#### Discussion on discrepancies and error sources

The comparison reveals that while the errors for pile head displacement, rotation, and shear force are within acceptable margins (4.8% to 8.2%), the discrepancy for the maximum bending moment is notably higher (approximately 24.9%). This divergence warrants a focused discussion on its potential origins, which are inherently linked to the simplifications of the analytical model versus the more detailed numerical simulation:

Limitations of the Linear Winkler Model in Capturing Local Stress Redistribution: The *m*-method, underpinned by the linear Winkler foundation assumption, is inherently a smoothed, global-scale approximation. It struggles to perfectly capture the highly localized and nonlinear stress redistribution that occurs in the immediate vicinity of the mechanical joint—a region of geometric and material discontinuity. The FEM, with its continuum modeling of soil and explicit joint representation, can more accurately resolve these complex local stress fields, leading to a different predicted magnitude and location of the maximum bending moment.

Effect of Joint Modeling and Mesh Sensitivity: In the finite element model, the joint region and the adjacent pile segments are discretized with a refined mesh. This allows for a more precise resolution of stress concentrations at the joint interface, which directly influences the bending moment profile. The analytical model, by contrast, represents the joint’s effect through a boundary condition (a rotational hinge) without simulating the local three-dimensional stress state. Part of the observed discrepancy can be attributed to this fundamental difference in modeling the joint’s mechanical influence on stress flow.

Inherent Simplification vs. Detailed Simulation: The core of the discrepancy underscores the trade-off between analytical tractability and numerical precision. The proposed *m*-method extension provides a closed-form, computationally efficient solution by idealizing a complex physical system. The 25% error in peak bending moment primarily highlights the limitation of this linearized approach in predicting the exact extreme value of a second-order derivative quantity (moment) in a discontinuous system, whereas it performs satisfactorily for displacement and rotation (zero and first-order derivatives).

Therefore, the observed error in bending moment is not anomalous but rather a systematic outcome of the model’s simplifying premises. It indicates that while the extended *m*-method reliably predicts the overall deformation pattern and force distribution trend of the pile, engineers should exercise caution when using its absolute bending moment values for final, detailed design of the pile section at the joint region. Its primary strength lies in conceptual understanding, preliminary design, parametric studies, and capturing the comparative behavioral shift between conventional and jointed piles, as demonstrated in Section “Comparative analysis of mechanical characteristics”.

### Comparative analysis of mechanical characteristics

**Fig. 9:**
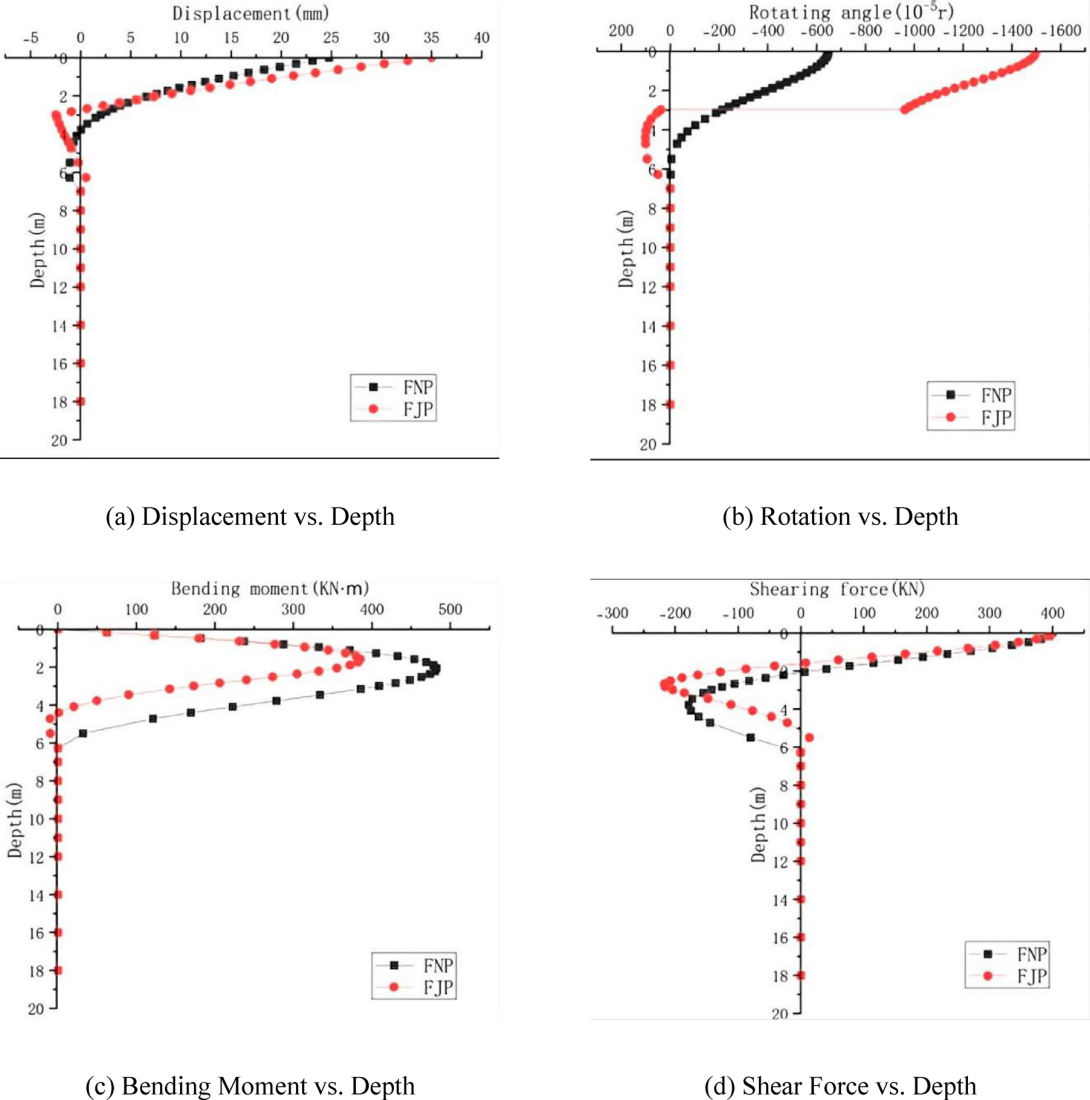
Comparison of key mechanical responses between the conventional single pile and the mechanically-jointed pile based on the *m*-method.

To ensure a fair and unambiguous comparison of the fundamental influence of the mechanical joint, the conventional single pile and the segmented mechanically-jointed pile are designed to be geometrically identical in the global dimensions. Specifically, both piles share the same total length, embedded depth, and cross-sectional dimensions. The sole difference lies in the presence (or absence) of the mechanical joint and the associated structural discontinuity. The *m*-method is employed to calculate the responses of both pile types under identical lateral loading and soil conditions. The results are shown in Fig. 9 and summarized in Table 5.

**Table 5:** Comparison of characteristic response values for different pile types.

Response parameter	Conventional single pile	Mechanically-jointed pile
Pile head displacement (mm)	24.77313	35.00511
Pile head rotation (× 10⁻^5^ rad)	− 648.40040	− 1499.81164
Max bending moment (kN·m)	482.24022	385.40234
Max negative shear force (kN)	− 178.58881	− 216.87968

Based on the comparison in Fig. 9 and Table 5, the following conclusions can be drawn:

1. Deformation Characteristics: Under the same boundary conditions at the pile base, the pile head displacement and rotation of the mechanically-jointed pile are significantly larger than those of the conventional single pile, with increases of approximately 30% and 55%, respectively. This is primarily due to the additional rigid body displacement of the upper pile segment caused by the opening of the connector gap.
  Assuming the upper short pile undergoes only rigid body rotation, the estimated increase in pile head displacement (Δ) when the connector rotation reaches its limit (0.01 rad) is:
  Δ = Rotation Limit × Upper Pile Length = 0.01 rad × 3 m = 0.03 m = 30 mm.
  The pile head displacement of the mechanically-jointed pile in this study is about 35 mm, more than 30 mm. This indicates that the pile-soil system has entered a stage of cooperative load-bearing and also validates the reasonableness of the horizontal load setting (400 kN), which is sufficient to engage the connector.
2. Internal Force Characteristics: Compared to the conventional single pile, the mechanically-jointed pile exhibits a approximately 20% reduction in maximum bending moment but a approximately 17% increase in maximum negative shear force. Furthermore, the locations of the maximum internal forces (bending moment and shear force) are closer to the pile head. This indicates that the mechanical connection alters the load-transfer mechanism of the pile, somewhat improving its bending resistance but increasing shear demand. The internal forces are also more concentrated in the upper part of the pile, suggesting a reduction in overall integrity.

## Conclusions

To investigate the mechanical behavior of free-headed, mechanically-jointed piles under lateral loading, this study derived an extended analytical framework based on the *m*-method and validated it through numerical simulations. The main findings are as follows:

1. The proposed theoretical method effectively predicts the overall deformation pattern and load-transfer trend of free-headed mechanically-jointed piles. Errors for pile head displacement (4.8%) and rotation (6.2%) are within a reasonable range for preliminary design. A larger discrepancy in the maximum bending moment (24.9%) is noted, highlighting the model’s limitation in capturing precise local stress concentrations near the joint, which is an inherent challenge for linearized analytical solutions.
2. Under identical free-head conditions, the mechanically-jointed pile exhibits significantly larger deformation than a conventional monolithic pile, with increases of approximately 30% in pile head displacement and 55% in rotation. This is primarily attributed to the additional rigid-body displacement caused by the rotation at the mechanical joint.
3. Compared to a conventional pile, the mechanically-jointed pile shows a distinct internal force redistribution: an approximately 20% reduction in the maximum bending moment but a 17% increase in the maximum shear force. This indicates a shift in structural demand, suggesting that design considerations for such piles should place greater emphasis on shear capacity and joint performance rather than bending resistance alone. It is emphasized that this change in internal force state does not directly equate to an enhancement in the system’s lateral bearing capacity, which is governed by broader serviceability or ultimate limit state criteria.

## Limitations and future work

### Limitations

The present study has several limitations that should be acknowledged:

1. The theoretical model relies on simplifying assumptions, including a linear soil response per the *m*-method and an idealized mechanical joint modeled as a plastic hinge with a fixed rotation limit $\varphi^{0}$. These may not fully capture nonlinear soil behavior or the joint’s complex constitutive relationship.
2. The validation scope is limited. The favorable agreement between the analytical and numerical results is established for a specific set of pile geometries and homogeneous soil conditions. Therefore, the quantitative conclusions regarding the degree of response changes may not be directly generalizable to other configurations without further verification.
3. Due to the lack of published experimental data for free-headed, mechanically-jointed piles, the validation in this study is primarily numerical. Direct benchmarking against physical test results is needed.

### Future work

To address these limitations and advance the research, the following directions are recommended:

1. Incorporate advanced soil models: Extend the analytical framework by integrating nonlinear soil models (e.g., *p-y* curves) to better capture soil behavior under larger deformations.
2. Characterize real joint behavior: Conduct dedicated experimental tests to obtain accurate moment-rotation relationships for mechanical joints and to study their performance under cyclic loading.
3. Perform comprehensive parametric studies: Systematically investigate the influence of key variables such as pile slenderness ratio, joint position, soil stiffness profile (*m* value), and load magnitude. This will delineate the method’s applicability and may lead to practical design guidelines.
4. Conduct sensitivity and reliability analyses: Quantify the influence of uncertain parameters, especially the joint rotation limit $\varphi^{0}$, on the system response to establish the robustness and reliability of the design method.

## Data Availability

The datasets generated and analysed during the current study are available in the Mendeley Data repository, [Liu, Tao (2025), “Calculated Coefficients for [Response of Free Headed Segmental Piles with Mechanical Joints to Lateral Loading]’s Formula Derivation”, Mendeley Data, V1, 10.17632/2yrf9s6h4g.1].
